# Spatial transcriptomic survey of human embryonic cerebral cortex by single-cell RNA-seq analysis

**DOI:** 10.1038/s41422-018-0053-3

**Published:** 2018-06-04

**Authors:** Xiaoying Fan, Ji Dong, Suijuan Zhong, Yuan Wei, Qian Wu, Liying Yan, Jun Yong, Le Sun, Xiaoye Wang, Yangyu Zhao, Wei Wang, Jie Yan, Xiaoqun Wang, Jie Qiao, Fuchou Tang

**Affiliations:** 1 Beijing Advanced Innovation Center for Genomics, Department of Obstetrics and Gynecology, College of Life Sciences, Third Hospital, Peking University, Beijing, 100871 China; 2 Biomedical Institute for Pioneering Investigation via Convergence and Center for Reproductive Medicine, Ministry of Education Key Laboratory of Cell Proliferation and Differentiation, Beijing, 100871 China; 3 State Key Laboratory of Brain and Cognitive Science, CAS Center for Excellence in Brain Science and Intelligence Technology; Institute of Brain-Intelligence Science and Technology Zhangjiang Laboratory (Shanghai), Institute of Biophysics, Chinese Academy of Sciences, Beijing, 100101 China; 4 University of Chinese Academy of Sciences, Beijing, 100049 China; 5 Shanghai Center for Brain Science and Intelligence Technology, Institute of Biophysics, Chinese Academy of Sciences, Beijing, 100101 China; 6 Key Laboratory of Assisted Reproduction, Ministry of Education, Beijing, 100191 China; 7 Beijing Institute for Brain Disorders, Beijing, 100069 China; 8 Beijing Key Laboratory of Reproductive Endocrinology and Assisted Reproductive Technology, Beijing, 100191 China; 9 Peking-Tsinghua Center for Life Sciences, Peking University, Beijing, 100871 China

## Abstract

The cellular complexity of human brain development has been intensively investigated, although a regional characterization of the entire human cerebral cortex based on single-cell transcriptome analysis has not been reported. Here, we performed RNA-seq on over 4,000 individual cells from 22 brain regions of human mid-gestation embryos. We identified 29 cell sub-clusters, which showed different proportions in each region and the pons showed especially high percentage of astrocytes. Embryonic neurons were not as diverse as adult neurons, although they possessed important features of their destinies in adults. Neuron development was unsynchronized in the cerebral cortex, as dorsal regions appeared to be more mature than ventral regions at this stage. Region-specific genes were comprehensively identified in each neuronal sub-cluster, and a large proportion of these genes were neural disease related. Our results present a systematic landscape of the regionalized gene expression and neuron maturation of the human cerebral cortex.

## Introduction

The adult brain of vertebrate animals has extensive capabilities due to its astonishing cell type diversity^1,2^ and precise arrangement of regional structures,^3^ especially in the cerebral cortex as it is the most evolved organ with the most complex functions in human. The cerebral cortex contains convoluted, layered gray matter that is only 2–3 mm thick in human but with several hundred square centimetres of surface area.^4^ Neurons residing in the gray matter are the basic unit in the system and possess outgoing axons that club together to form the white matter of the cerebral cortex. Neurons located in different cortical layers and regions project to their specific destinations where they can receive and release signals by transmitting neurotransmitters to feel and control.^5–7^ Previous classifications for neurons were mainly based on their morphological, chemical, and electrical properties. As these properties are controlled genetically, neuron sub-cluster classifications have been defined by distinct molecular characteristics in recent studies.^8–12^

The enormous diversity of neurons with precise framework comes from genetically committed neural stem cell (NSC) and progenitor pools.^13,14^ Apart from the diverse neurons, progenitor pools produce more abundant glial cells including astrocytes and oligodendrocytes.^2^ These glial cells do not transmit signals like neurons, but they constitute the environment to chaperon the neurons and shape the neuronal network,^14,15^ and their dysfunction is associated with many neural system diseases.^16–18^ Although we have known that the neuronal and glial lineages share the same origin, the genetic determinants diversifying the neural progenitors into neuronal or glial specification are still not fully understood.

As the major architecture of the adult brain is almost established at the embryonic stage, dissecting the cell complexity and specific regional features of the developing cortex is a promising strategy for studying the functional specialization of the cerebral cortex. Previous studies, which have analyzed temporal and spatial neural development in rodent, human, and non-human primate brains, and have uncovered specific regional and temporal molecular characteristics of brain development, were almost based on bulk RNA-seq analysis.^19–24^ The molecular profiles of each structure can be unveiled by analyzing micro-dissected cortical tissues. However, such assessments are far from revealing the detailed mechanisms of cerebral cortex organization, as dissected structures are still composed of multiple cell types.

Single-cell transcriptome analysis may provide more precise information according to current progress, especially on cell type diversities,^8–11,25–31^ but barely approach the regional information to reveal the transcriptional landscape of the entire human embryonic cerebral cortex at single-cell resolution. In this study, we collected single cells for transcriptome analysis from different regions of the entire human cortex at 22 and 23 weeks post-conception (22 W and 23 W) and supplied the first data source to lay the ground for understanding the cell type constitution and molecular differences of regional development in the whole human cerebral cortex at the mid-gestational stage.

## Results

### Global clustering and identification of the single cells

**Fig. 1:**
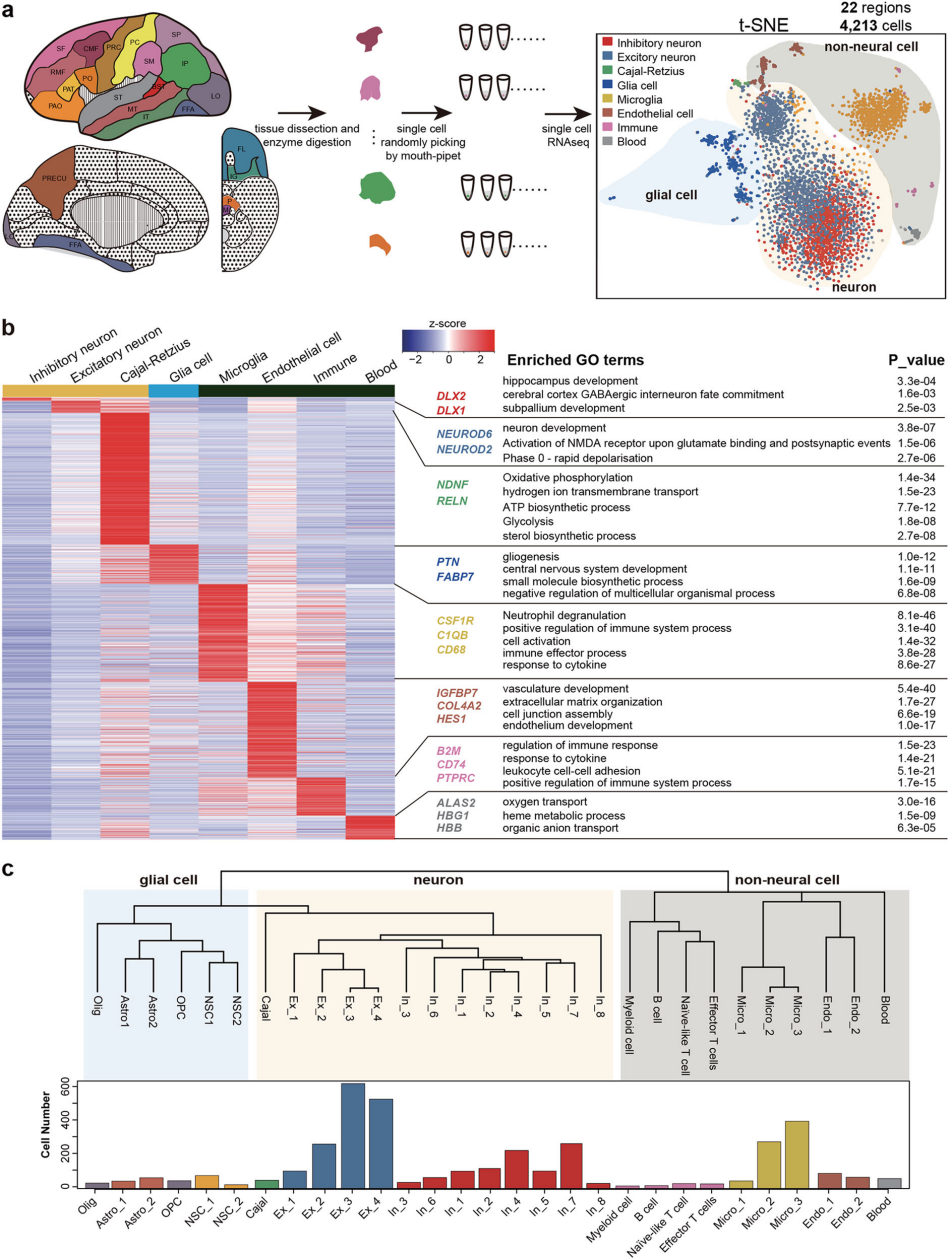
Cell type classification in human embryonic cerebral cortex. **a** The schematic diagram displaying the dissection of embryonic brain and how we obtained the single cell transcriptome data in 22 regions (for the abbreviations, see Supplementary information, Table S1). t-SNE showed the cell types identified with all the single cells and the shadows mark different general cell types: the blue shade indicates glial cell, the yellow shows neuron and the gray shows non-neural cell that is not supposed to be produced by neural stem cell. **b** Heatmap displaying the DEGs that were cell type specific in our analysis. The enriched biological processes for each gene group were shown in the right. Classical cell type marker genes were labeled. **c** Dendrogram showing the relationships of all the 29 sub-clusters and the histogram displaying the cell number in each sub-cluster

To detect the molecular distributions of 20 major anatomical cortical regions together with the medulla and the pons, we picked single cells as summarized in Supplementary information, Table S1. A total of 4,213 single cells from the cerebral cortex of a 22 W embryo and two 23 W embryos were analyzed. An average of 1.3 × 10^6^ mappable reads were generated for each cell, and on average, 4,318 genes were detected in each individual cell. We performed the t-distributed stochastic neighbor embedding (t-SNE) analysis to explore the diversity of all these cells. The even mixture of cells from different embryos in each cluster on the t-SNE plot reflected negligible individual variance or a batch effect for each cell type (Supplementary information, Figure S1a). Based on the global expression patterns, all cells were classified into three major groups, namely, neurons, glial cells and non-neural cells (Fig. 1a).

Further, each major group was divided using a random forest algorithm and we obtained eight major cell types, including inhibitory neurons, excitatory neurons, Cajal-Retzius cells, glial cells, microglia, endothelial cells, immune cells, and blood cells (Fig. 1a and Supplementary information, Table S2). Classical markers were specifically detected in the corresponding clusters of cells (Supplementary information, Figure S1b). In total, 1,672 differentially expressed genes (DEGs) were obtained among these major cell types using Seurat analysis. Gene ontology (GO) analysis also showed that each type of cell specifically expressed genes involved in the corresponding biological processes, as expected (Fig. 1b and Supplementary information, Table S3).

Neuronal cells (inhibitory neurons, excitatory neurons and Cajal-Retzius cells) accounted for 66% of the cells in the 22/23 W cerebral cortex, whereas glial cells (which could be further divided into NSCs, oligodendrocyte progenitor cells (OPCs), oligodendrocytes, and astrocytes) only accounted for 8% of the cells (Fig. 1c and Supplementary information, Figure S1c). This cellular distribution is markedly different from that found in the gray matter of the adult cerebral cortex, where glial cells are thought to outnumber neurons by 1.5 times.^32^ However, co-immunostaining for NEUROD1 and GFAP validated the much higher abundance of excitatory neurons than astrocytes in different cortical regions at this embryonic stage (Supplementary information, Figure S1d). Thus, we deduced that the preferential expansion of glial cells over neurons in the cerebral cortex occurs after 23 weeks post-fertilization in humans. Very different from the cortical regions, regions in the inferior surface such as the insular gyris (IG) and medulla contain more glial cells (Supplementary information, Figure S1e). More microglia were observed in the medulla than in the IG region, while these microglia in the medulla seemed less developed than those in the IG regions as the former are ameboid whereas the latter start to develop ramified form (Supplementary information, Figure S1e). This indicates that microglia development was unsynchronized in different brain regions. The vast majority of the cells in the cortex were in a quiescent/post-mitotic state at this embryonic stage, except that ~40% of the NSCs were still actively dividing (Supplementary information, Figure S1f).

### Neuron sub-clusters in the human embryonic cortex

**Fig. 2:**
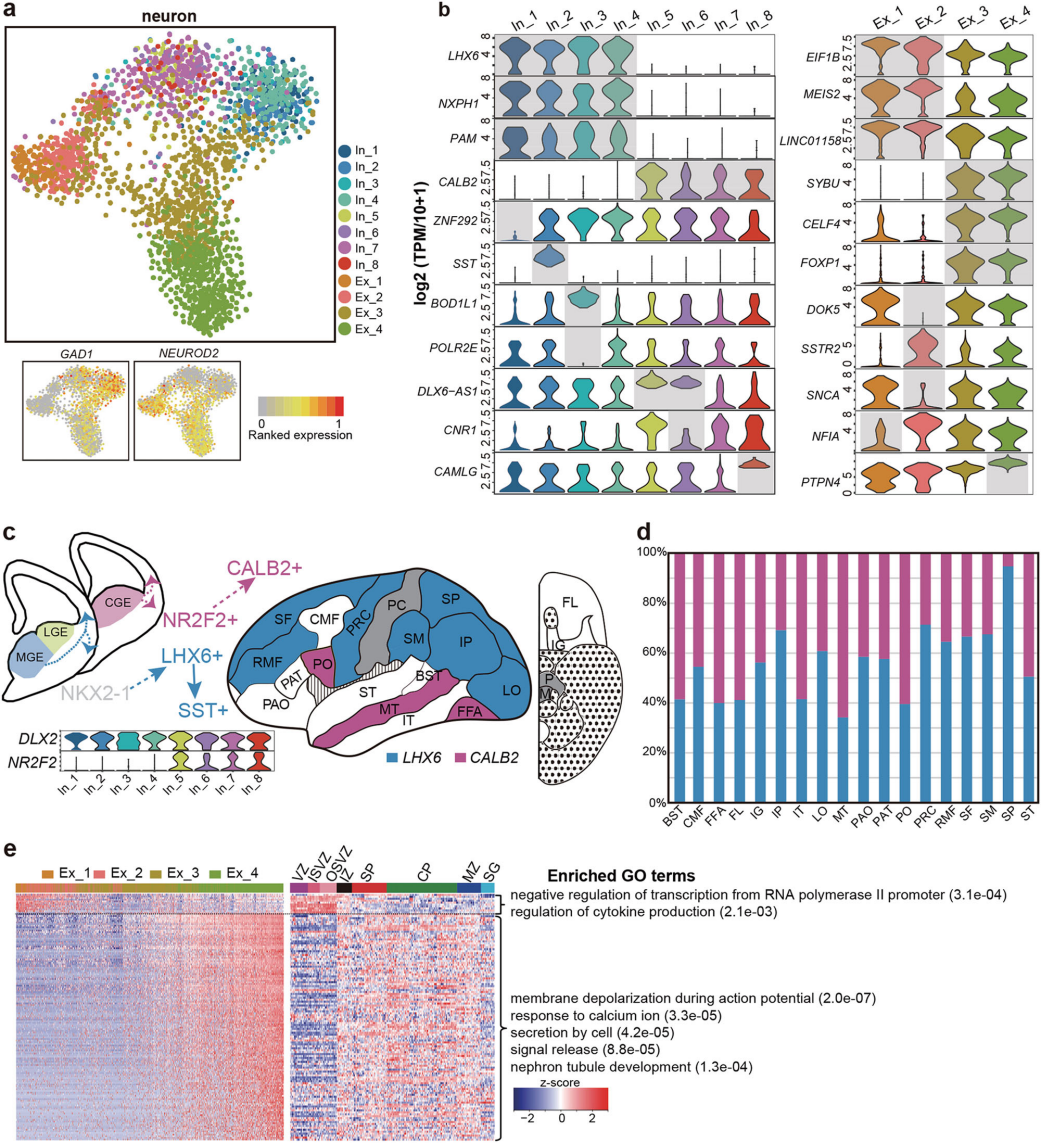
Neuron cell sub-clusters in human embryonic cerebral cortex. **a** t-SNE map showing the subtypes of all inhibitory and excitatory neurons. The inhibitory neuron could be subdivided into eight clusters, and all of those are *GAD1* positive. The excitatory neurons are *NEUROD2* positive and could be subdivided into four clusters. *In* inhibitory neuron, *Ex* excitatory neuron. **b** Violin plot showing the DEGs of subgroups with inhibitory neuron (left) and exicitatory neuron (right), respectively. **c** Schematic diagram describing where different types of inhibitory neurons are generated and how they migrated. Violin plots show expression levels of interneuron progenitor genes *DLX2* and *NR2F2* in each subgroups. The cortex landscape shows the dominant inhibitory neuron types in each region. Blue indicates that over 60% inhibitory neurons in the corresponding region are in *LHX6* subtype and the purple indicates that over 60% inhibitory neurons are in *CALB2* subtype. White indicates that both subtypes of inhibitory neurons make up 40%–60% of the sum. Regions of gray color are detected with <15 inhibitory neurons. **d** The accurate percentages of inhibitory neurons belonging to *LHX6* and *CALB2* subgroups in regions colored with blue, purple, and white. **e** Left, heatmap showing the genes positively and negatively regulating excitatory neuron maturation, respectively. The color bars at the top represent cells from different clusters, which are arranged in a pseudotime order from immature to mature neurons. Middle, expression of putative excitatory neuron maturation regulating genes in the structures studied by Miller et al.^20^ (sample 12566). Right, enriched biological processes for down-regulated and up-regulated genes in excitatory neuron maturation. *VZ* ventricluar zone, *ISVZ* inner subventricular zone, *OSVZ* outer subventricular zone, *IZ* intemediate zone, *SP* subplate zone, *CP* cortical plate, *MZ* marginal zone, *SG* subpial granular zone

To further reveal the subpopulations of each major cell type, we performed a more detailed analysis of each cell type with the random forest algorithm. A total of 968 inhibitory neurons were further divided into 8 sub-clusters, with each sub-cluster characterized by unique marker genes such as neural peptides (*NXPH1*, *SST*), enzymes (*PAM*), important calcium-binding proteins (*CALB2*, *CAMLG*), transcription factors (TFs) (*LHX6*), and non-coding RNAs regulating GABAergic cell fate (*DLX6-AS1*) (Fig. 2a). Inhibitory neuronal subgroups 1-4 highly expressed *LHX6* (Fig. 2b); these subgroups are thought to be generated in the medial ganglionic eminence (MGE), a major source of the GABAergic population in the cerebral cortex.^33^ These cells expressed *LHX6* together with *NXPH1* and *PAM*, and accounted for 50.9% of all the inhibitory neurons in the cortex. Furthermore, 24.9% of the *LHX6*^+^ progenitors developed into a somatostatin (SST)-expressing population (subgroup In_2). We identified a *BOD1L1*^+^ subgroup among the inhibitory neurons (Fig. 2b,c). This subgroup of cells did not express *POLR2E*, which encodes a subunit of RNA polymerase II. The other 49.1% of the inhibitory neurons (subgroups 5-8) were *CALB2* positive. These cells expressed both *DLX2* and *NR2F2* (encoding COUP-TFII) (Fig. 2c), indicating that they may originate from the caudal ganglionic eminence (CGE) and then migrate into the cortex.^34^ By calculating the proportions of *LHX6*^+^ and *CALB2*^+^ inhibitory neurons in each cortical region, we found that *LHX6*^+^ neurons are relatively enriched in the superior part of the cerebral cortex, whereas the *CALB2*^+^ neurons are enriched in the inferior regions (Fig. 2c, d).

The 1,625 excitatory neurons were further subdivided into 4 groups, and groups 1 and 2 showed higher expression of more immature genes, such as *EIF1B*, *MEIS2*, and *LINC01158*^35^ (Fig. 2b). The ortholog of *LINC01158* in mouse, *Pantr1*, regulates the differentiation of neuronal progenitors, whereas its paralog *Pantr2* regulates the expression of *Pou3f3* and maintains the proliferation of progenitor cells in the developing cortex.^36,37^ The expression pattern of *LINC01158* indicated a different role in humans compared with that in rodents. The other two subgroups were more mature, as these groups highly expressed *SYBU* and *CELF4*. *FOXP1*, a TF important for medium spiny projection neurons, was also detected in the excitatory neuron subgroups 3 and 4 in the cerebral cortex.^38^ Based on a pseudotime analysis, all the excitatory neurons were arranged according to a developmental course. Subgroup 1 (Ex_1) to subgroup 4 (Ex_4) cells ranked sequentially from earlier to later in the pseudotime course, as expected (Fig. 2e, left). Genes down-regulated during the differentiation course of the excitatory neurons were mainly associated with negative regulation of RNA polymerase II transcription and cytokine production. These genes were more enriched in germinal structures (VZ, ISVZ, and OSVZ), as reported by Miller et al.^20^ (Fig. 2e and Supplementary information, Figure S2a). In contrast, the positively regulated genes were enriched in synaptic functions, such as membrane depolarization during action potentials, and showed higher expression levels in the upper structures of the cortex (IZ to MZ). These genes were enriched in neuron-related functions, such as membrane depolarization during action potential and signal release (Fig. 2e and Supplementary information, Figure S2a). These findings suggest that the molecular mechanism underlying the excitatory neuron differentiation could be revealed by scRNA-seq.

**Fig. 3:**
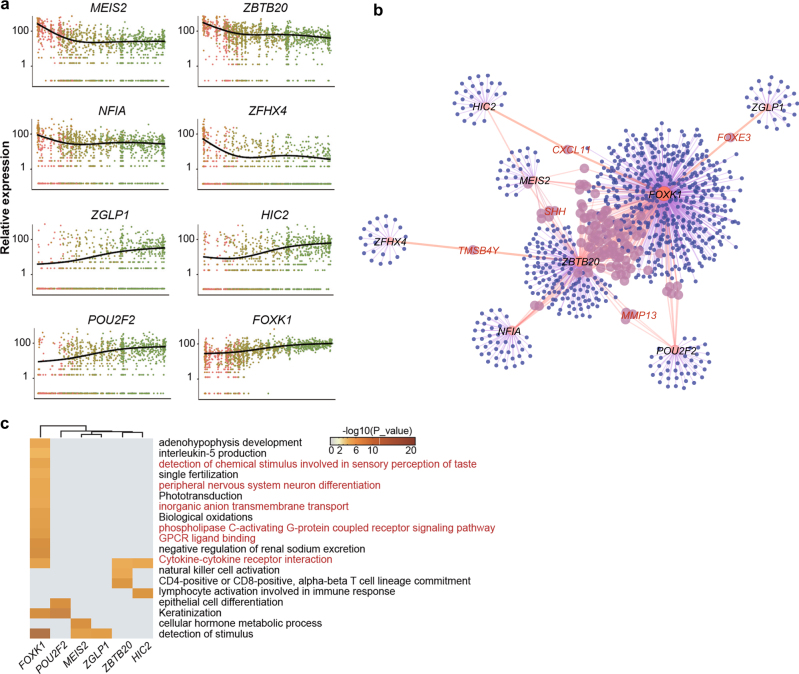
Regulation network of the transcription factors in excitatory neuron differentiation. **a** The expression changes of TFs negatively (top row) and positively (bottom row) regulating the maturation of excitatory neurons along the pseudotime. **b** The transcription networks of the TFs regulating excitatory neuron maturation. Top 1,000 target genes for each TF were listed in Supplementary information, Table S4. **c** Enrichment analysis on target genes for each TF shown in **b** and **c**. No enriched terms for NFIA and ZFHX4. Terms colored in red are known pathways quite related to neural development

Since subgroups Ex_1 to Ex_4 showed a developmental relationship based on pseudotime analysis, we picked the TFs that potentially regulated the maturation of excitatory neurons from DEGs among these four groups. Four TFs (*ZGLP1*, *HIC2*, *POU2F2*, and *FOXK1*) potentially positively regulated the maturation of excitatory neurons and another four TFs (*MEIS2*, *ZBTB20*, *NFIA*, and *ZFHX4*) potentially negatively regulated the maturation of excitatory neurons in the developing human cortex, respectively (Fig. 3a). We further analyzed the regulation network of these eight TFs (Fig. 3b and Supplementary information, Table S4) by analyzing the genes co-expressed with the TFs and extracted the top 1,000 links showing positive correlation with each TF. We found that *FOXK1* was a candidate core TF regulating the maturation of excitatory neurons since it showed the strongest co-regulation patterns with potential target genes. Analysis of biological process enrichment in the target genes of each TF further suggested that *FOXK1* participated in multiple processes of neuronal maturation (Fig. 3c).

### Comparison of human embryonic neurons to adult neurons in the cortex

**Fig. 4:**
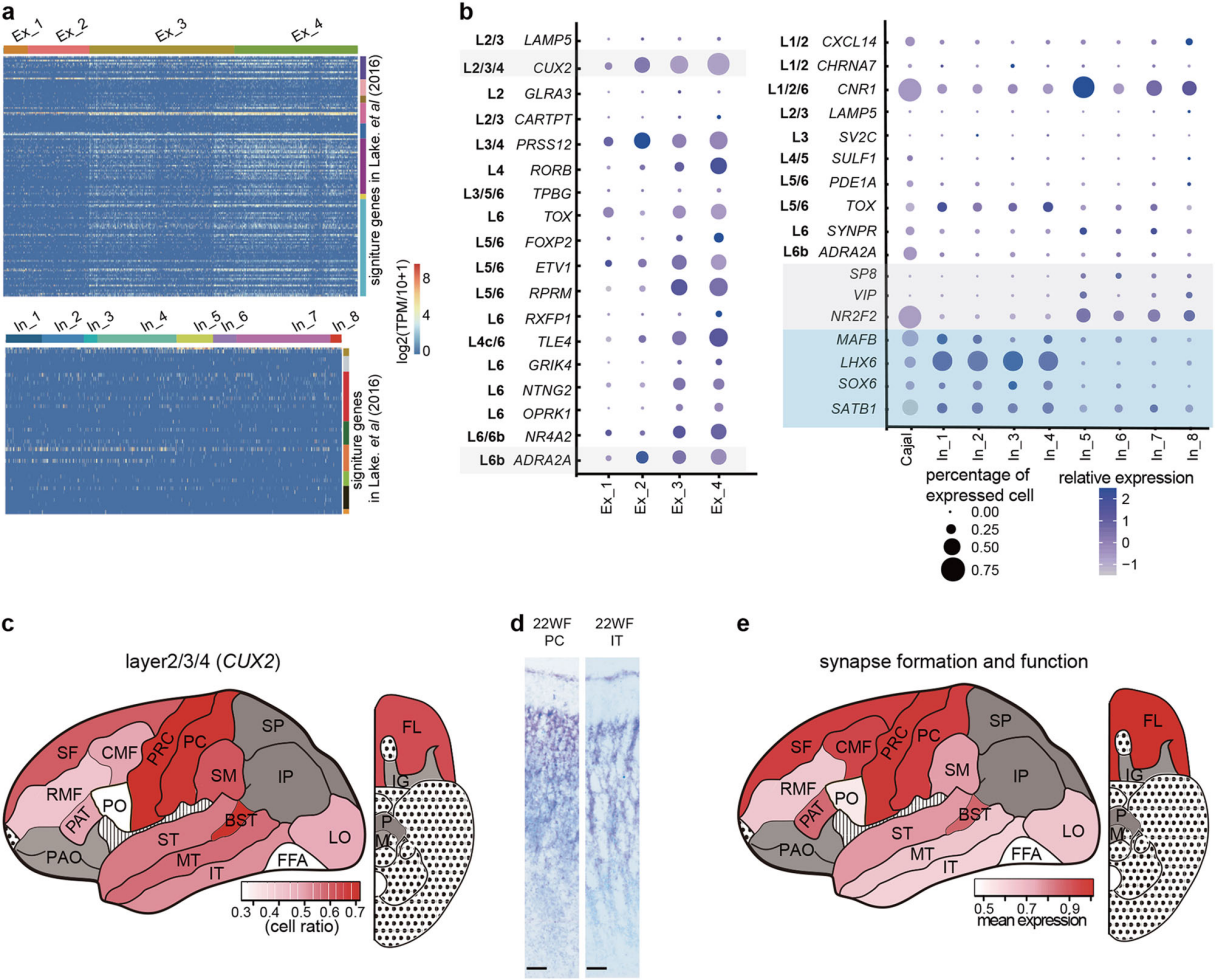
Maturation degree of embryonic neurons is different from the adult neurons. **a** Heatmap showing the expression of marker genes identified in the adult neuron subtypes. **b** Expression of layer markers in different sub-clusters of excitatory neuron (left) and inhibitory neuron (right). **c** Landscape showing the maturity level of each region in the cortex measured by *CUX2*-positive excitatory neuron ratio. Regions of gray color are ruled out as they are detected with < 50 neurons. **d** In situ hybridization of *CUX2* in PC and IT regions of a 22WF embryo. Scale bar, 100 μm. **e** Landscape showing the maturity level of each region in the cortex measured by expression level of synapse formation and function related genes

Lake et al. revealed the molecular signatures of neurons in different cortical regions of the human adult brain.^26^ We wondered whether these signatures were already formed in the mid-gestational embryonic cortex. First, we analyzed the expression levels of excitatory and inhibitory neuron sub-cluster markers identified in the adult cortex (Fig. 4a). Approximately half of the excitatory sub-cluster markers were expressed in the embryonic excitatory neurons; these markers could be used to discern only the Ex_1/2 from the Ex_3/4 subgroups. Few of the inhibitory sub-cluster markers were detected in the embryonic inhibitory neurons, perhaps because both the excitatory and inhibitory neurons at this embryonic stage were not fully developed with functional projections, and most of these neurons were still progenitors during migration. Further analysis of the expression levels of the layer markers also showed no patterns of these adult neuron markers on the embryonic neuronal sub-clusters for either excitatory or inhibitory neurons (Fig. 4b). Only the more mature sub-clusters of excitatory neurons, Ex_3 and Ex_4, showed a broad distribution across layer 6b to layer 2 and these cells expressed markers of multiple layers (Supplementary information, Figure S2b). We suspected that these migrating neurons express multiple layer signatures at 23 W, and later, when they have arrived at their final destination, they will mature and express marker genes specific for the layer that they migrate into and develop full projections.

We noticed that the expression level of upper layer marker *CUX2*^39,40^ could clearly specify the four sub-clusters of excitatory neurons and the more mature neurons showed higher expression of *CUX2* (Fig. 4b). Thus, *CUX2* could be used to evaluate the maturation degree of each region in the whole cerebral cortex. According to the *CUX2*^*+*^ cell ratio in each region, we found that the neurons in the pre-central cortex (PRC, also known as the primary somatomotor cortex) and the post-central cortex (PC, also known as the primary somatosensory cortex) were more mature than the neurons in other cortical regions (Fig. 4c). RNA in situ hybridization of *CUX2* was performed for the PC and the inferior temporal cortex (IT) regions, which indeed confirmed more *CUX2*^*+*^ cells in the PC region (Fig. 4d). Similar mature patterns were revealed when we analyzed the expression levels of synapse formation and function related genes as measures of neuron maturity in each cortical region (Fig. 4e).

**Fig. 5:**
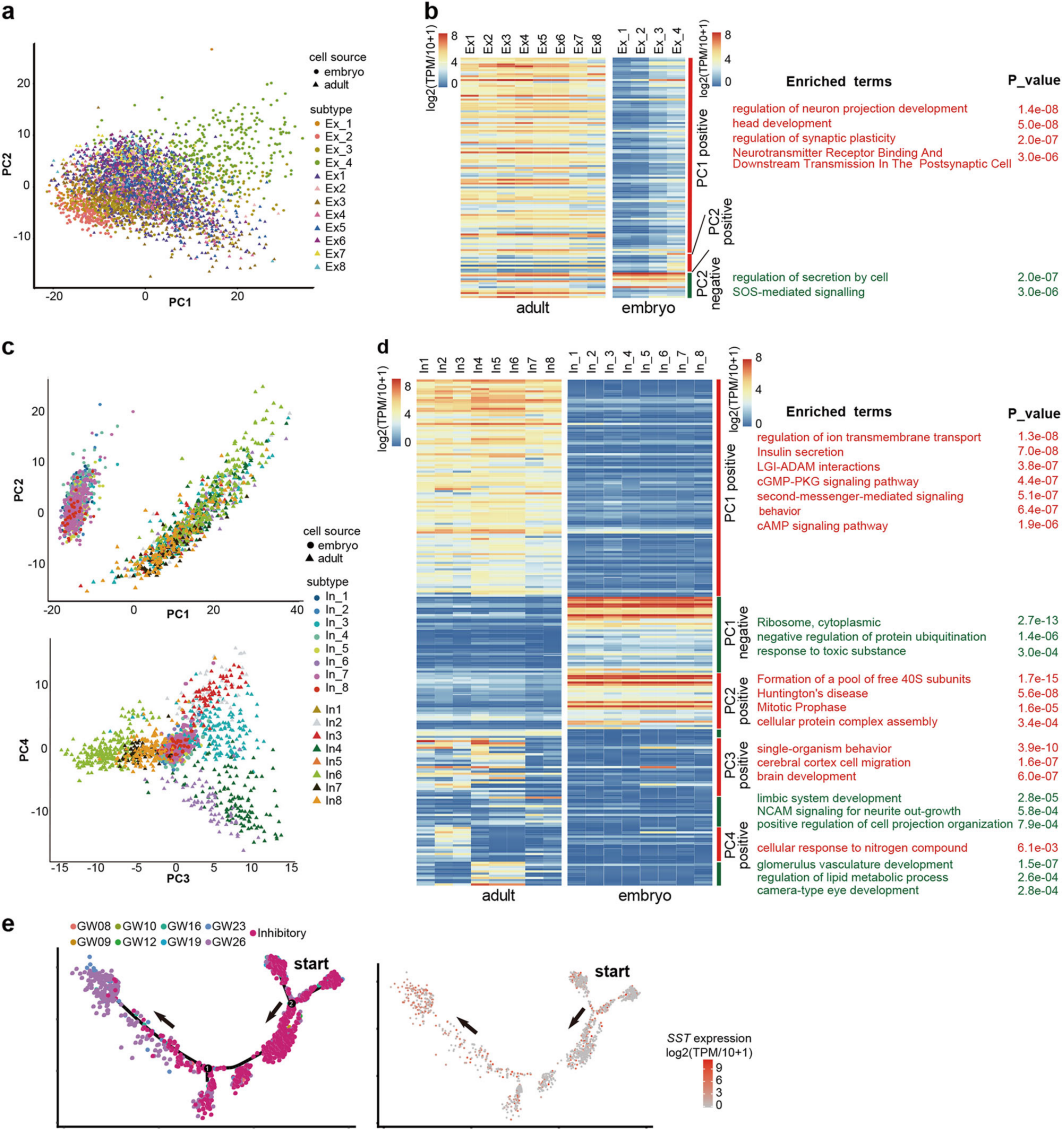
Comparison of embryonic neuron sub-clusters to adult ones. **a** PCA plot of both excitatory neuron sub-clusters and adult ones. **b** Heatmap of expression of the top genes in the PCs corresponding to panel (**a**) and the enriched terms for each PC gene set. Red bar indicates the genes positively correlated in each PC and the green bar indicates the negatively correlated genes in each PC. **c** PCA plot of both inhibitory neuron sub-clusters and adult ones. **d** Heatmap of expression of the top genes in the PCs corresponding to panel (**c**) and the enriched terms for each PC gene set. **e** Monocle analysis of inhibitory neurons together with those identified in developing pre-frontal cortex. *SST*^+^ cells show up randomly on the pseudotime

To obtain further insight into the differences between embryonic and adult cortex neurons, we compared our single-cell data with those of the adult neurons by principal component analysis (PCA) after removing batch effect (Fig. 5a). The findings showed that the heterogeneity of the excitatory neurons at mid-gestation stage was already comparable to that of the adult brain. Both PC1 and PC2 reflected the differences between immature and mature embryonic neurons, and the enriched terms were also associated with projection development and synaptic plasticity (Fig. 5b). The results for inhibitory neurons were very different. PC1 genes clearly separated the embryonic inhibitory neurons from the adult neurons (Fig. 5c). The adult inhibitory neurons highly expressed PC1 positive direction genes, which were enriched in neuron signaling genes, whereas the embryonic inhibitory neurons specifically expressed PC1 negative direction genes, which were associated with cellular metabolism. The PC3 and PC4 genes mainly reflected the differences among the subgroups of adult inhibitory neurons, whereas the embryonic inhibitory neurons exhibited no significant differences on these two axes (Fig. 5d). We inferred that this difference might exist because the mid-gestation inhibitory neurons were still immature and had not differentiated into subtypes comparable to those in the adult cortex. Since the *SST*^+^ inhibitory subgroup is classified as a sub-cluster at this stage, we wondered whether this subtype of neuron matures earlier than other subtypes of inhibitory neurons. We combined our inhibitory neurons with those identified in the developing pre-frontal cortex^26^ and performed pseudotime analysis. Inhibitory neurons at 22–23 W in this study mainly located from early to the middle developing stages on the pseudotime path (Fig. 5e). The *SST*^+^ neurons randomly appeared in the whole developing course of the pseudotime path, indicating that they were not more mature compared to other inhibitory neurons. Thus we inferred that the *SST* expression should not be of the same function as those in adult neurons.

**Fig. 6:**
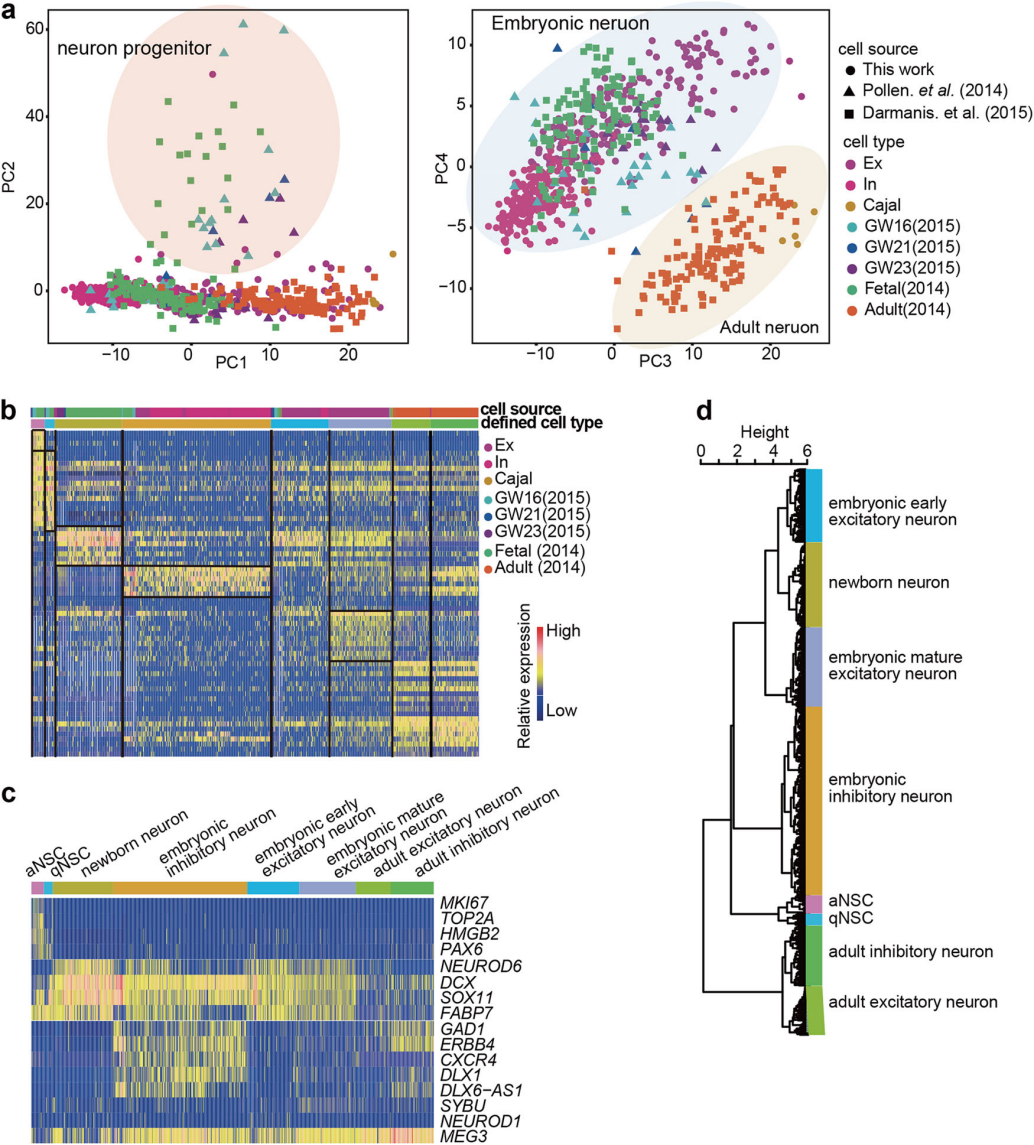
Comprehensive analysis on cell types of human cortex. **a** PCA of single neuronal cells from different data sets. The PC2 clearly separates progenitors from the differentiated cells, and PC3 and PC4 separate embryonic cells from the adult cells. **b** Heatmap showing the clustering of cells from different data sets and group-specific gene expression. **c** Classical marker gene expression in each identified cell group. **d** Unsupervised clustering of the 8 cell groups showing the distances between the cell groups

To more thoroughly analyze the cell types of the human cortex, we also compared our data with the single-cell data from Pollen et al.^27^ and Darmanis et al.^11^ We analyzed all the single-cell samples by PCA after batch effect correction using mutual nearest neighbor (MNN) algorithm in Scran package.^41,42^ The PC2 positive direction genes were enriched for the neuron progenitor-specific genes, especially for those at early embryonic stage (Fig. 6a). PC3 and PC4 clearly separated the adult cells from the embryonic cells, except for the Cajal-Retzius cells identified in our study, which mixed well with the adult cells. Thus, Cajal-Retzius cells were probably the cells that kept the most similar expression signatures from mid-gestation stage to adult. All the cells could be clustered into 8 groups, each showing a specific gene expression pattern (Fig. 6b). According to the marker gene expression signature for each group, both the actively dividing neural stem cell group and the quiescent neural stem cell group mainly consisted of GW16, GW21, and fetal neurons (Fig. 6b, c). The differences between adult excitatory neurons and inhibitory neurons were milder than those between the embryonic excitatory neurons and inhibitory neurons. The NSCs and adult neurons were the most unique cell types. Unsupervised clustering through Pearson distance calculation among these 8 groups revealed that the longest distance for the cell groups was between the adult neurons and the NSCs (Fig. 6d).

### Sub-clusters of non-neuronal cells in the human embryonic cortex

Glial cells were further subdivided into six sub-clusters (Supplementary information, Figure S3a and b). The two sub-clusters expressing *VIM* were NSCs (NSC_1 and NSC_2), and NSC_2 was more active in the cell cycle. These NSCs showed a glial fate preference to differentiate into astrocytes (marked by *AQP4*) and OPCs (marked by *PDGFRA*), and further into oligodendrocytes (marked by *MOBP)* (Supplementary information, Figure S3b and c). The non-neural cells were further divided into three microglial sub-clusters (marked by *CD68*, Micro_1, Micro_2, and Micro_3), four immune sub-clusters that were further identified as B cells (marked by *CD52*, *CD79A/B*), myeloid cells (marked by *LYZ*, *CSTA*), naïve-like T cells (marked by *IL7R*, *TCF7*), and effector T cells (marked by *NKG7*, *CST7*). There were two endothelial sub-clusters (marked by *SPARC*, Endo_1 and Endo_2) and one blood cell sub-cluster (marked by hemoglobin genes such as *HBG1*) (Supplementary information, Figure S4a and b). Cell cycle analysis of these subgroups revealed that NSC_2 and Micro_1 cells were in an actively dividing state (Supplementary information, Figures S3d and S4c).

In total, 29 sub-clusters of cells were identified in the human 22–23-week embryonic cerebral cortices, and neurons accounted for the majority of them. The more mature cell types, such as Cajal-Retzius cells and oligodendrocytes, expressed more genes and contained more abundant transcripts in each individual cell (Supplementary information, Figure S5). Similarly, the gene and transcript numbers increased along with the maturation of excitatory neurons. This effect was likely due to the increased transcription activity during the maturation of excitatory neurons (Fig. 2e and Supplementary information, Figure S2).

### Spatial differences for different types of cells in the developing human cortex

**Fig. 7:**
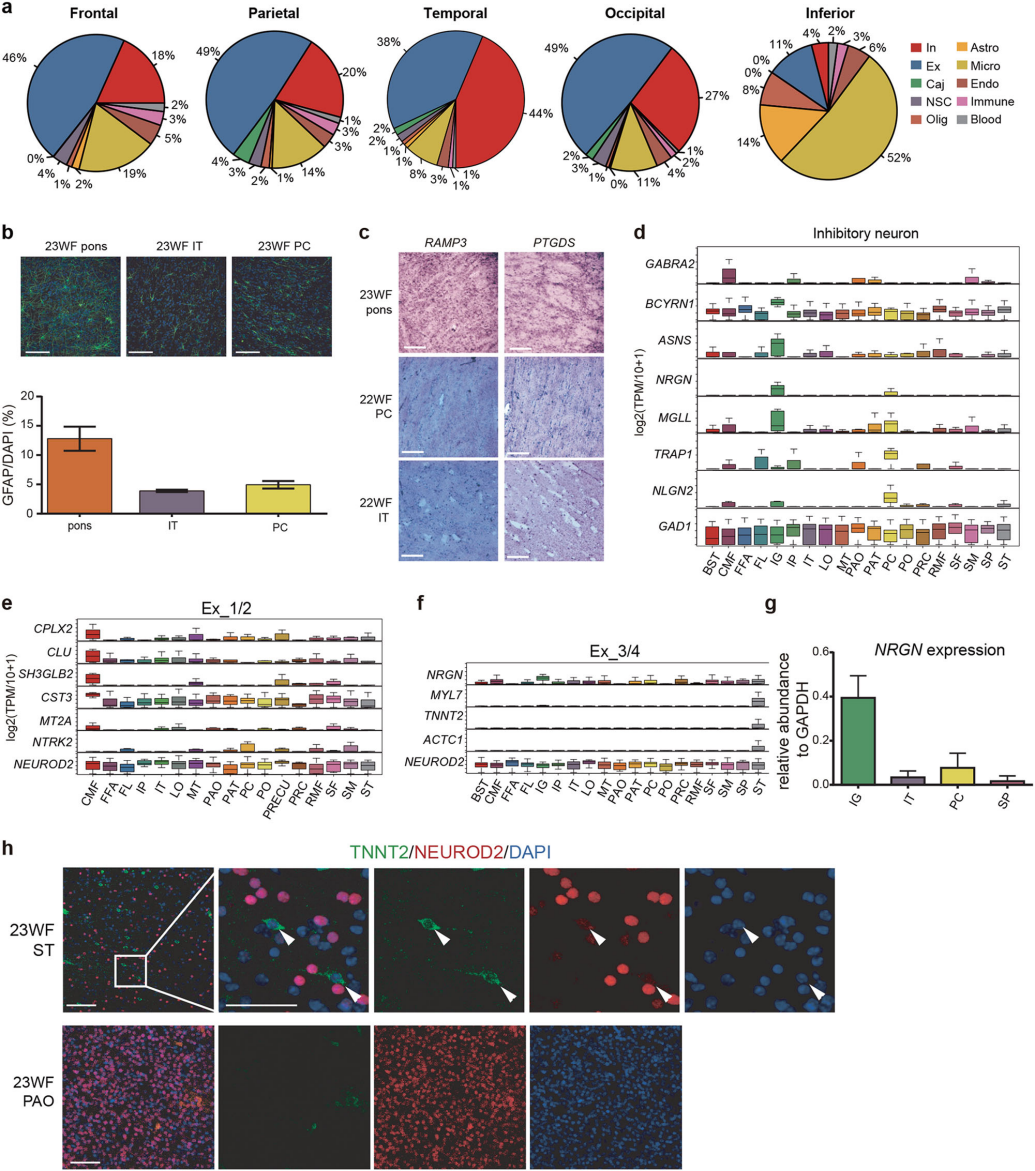
Spatial differences in the developing human cortex. **a** Pie chart displaying the cell type constitution in the four cerebral lobes and in the inferior region of cerebral cortex. **b** Immunofluorescence of GFAP in the pons, PC and IT regions of a 23 W female sample. The statistics of GFAP^+^ cell ratio in each region are shown in the histogram. Scale bar, 50 μm. **c** In situ hybridization of astrocyte genes *RAMP3* (Astro_1) and *PTGDS* (Astro_2) showing higher abundance of the two subtypes of astrocytes in pons than that in IT and PC regions. Moreover, the PC shows the lowest astrocyte density. Scale bar, 150 μm. **d–f** DEGs across all cerebral cortex regions that are detected with more than 5 inhibitory neurons (**d**), immature excitatory neurons (Ex_1/2, **e**) and mature excitatory neurons (Ex_3/4, **f**). *GAD1* and *NEUROD2* are the housekeeping control for inhibitory and excitatory neurons, respectively. **g** RT-qPCR of *NRGN* in IG, IT, PC, and SP regions. The *NRGN* abundance in each region was normalized by *GAPDH*. **h** Validation of excitatory neurons expressing myocardial protein TNNT2 in the 23WF ST region by immunofluorescence. The PAO region is displayed as a negative control. TNNT2 antibody was tested in the cadiomyocytes as shown in Supplementary information, Figure S6c. Scale bar, 50 μm

The spatial position recorded for each cell provided valuable information for investigating the regional differences in the embryonic cerebral cortex in terms of regional cell type diversity. The four main regions in the cortex, the frontal lobe, the parietal lobe, the occipital lobe, and the temporal lobe, consisted of more neurons (Fig. 7a), whereas regions in the inferior surface contained many more glial cells. We particularly observed a high abundance of astrocytes in the pons (Fig. 7a and Supplementary information, Figure S6a). Immunostaining for GFAP to detect cells from the pons, the IT and PC regions showed the most astrocytes in the pons. Impressively, the astrocytes in the pons showed more mature morphology, with longer processes than those in the PC and IT regions (Fig. 7b). To determine whether there was a distribution difference between the two sub-clusters of astrocytes, we performed in situ hybridization for *RAMP3* (marking the Astro_1 sub-cluster) and *PTGDS* (marking the Astro_2 sub-cluster) on the pons and cortical regions. Both sub-clusters were much more abundant in the pons than those in the PC and IT (Fig. 7c). We also noticed more excitatory neurons in regions belonging to the frontal lobe and more inhibitory neurons in the regions belonging to the temporal lobe (Fig. 7a and Supplementary information, Figure S6a). To validate the distribution differences of the cerebral cortex, we did RT-qPCR to compare the expression levels of *NEUROD2* and *GAD1* in the PC and IT regions. In agreement with the sequencing data, the PC region showed higher expression of *NEUROD2*, whereas the IT region showed higher expression of *GAD1* (Supplementary information, Figure S6b). Moreover, the immunostaining of NEUROD2 in PC and IT regions also showed relatively more excitatory neurons in the PC (Supplementary information, Figure S6c).

We next questioned whether there were differences in cell heterogeneity among different regions. We extracted regions with over 50 single cells analyzed by RNA-seq for specific neuron sub-clusters and calculated the correlation coefficients between individual cells within each region. The gene expression heterogeneity of both excitatory and inhibitory neurons in the frontal lobe was significantly lower than that of other regions for each neuron sub-cluster (Supplementary information, Figure S6d). In contrast, the excitatory neuron sub-clusters in each region were more homogeneous than the inhibitory sub-clusters in the same region, which was compatible with the rich diversity of interneurons in mature human cortex and mouse cerebral cortex.^8, 9,11^

We next analyzed DEGs among the cortical regions based on different neural sub-clusters. For inhibitory neurons (Fig. 7d), we found that the GABA receptor *GABRA2* showed region-specific expression. The neuronal functional genes *MGLL*, *TRAP1*, and *NLGN2*^43–45^ were more specifically expressed in the PC, indicating that regional DEGs may be involved in the formation of region-specific functions of the corresponding regions. We also found that Ex_1/2 neurons played a special role in the caudal middle frontal lobe (CMF) (Fig. 7e). Neuronal progenitors in this region highly expressed several genes related to neural diseases. For example, *CLU* and *CST3* are related to Alzheimer’s disease.^46,47^ Other region-specific genes such as *NTRK2*, which was highly detected in PC and supra-maginal regions, was reported to regulate the survival of neurons, and its down-regulation may cause autism.^48^ The BDNF/TrkB (TrkB also known as NTRK2) signaling pathway was also reported to be important in memory and learning during neural development.^49^ *NRGN* showed regionally differential expressions in both inhibitory and mature excitatory neurons but barely detectable expression in the Ex_1/2 neurons (Fig. 7d, f and Supplementary information, Figure S6e). RT-qPCR showed enriched expression of *NRGN* in the IG region, consistent with the single-cell RNA-seq data (Fig. 7g). Unexpectedly, we identified several myocardial genes, such as *MYL7*, *TNNT2*, and *ACTC1*, which were expressed in the ST (Fig. 7f). We observed that TNNT2 co-localized with the neural marker NEUROD2 in individual cells in both the 22 W and 23 W ST regions at the protein level (Fig. 7h and Supplementary information, Figure S6f). As a control, we did not find this group of cells in the pars orbitalis region (PAO) of the 22 W and 23 W embryos.

### Expression pattern and co-expression networks of autism risk genes

**Fig. 8:**
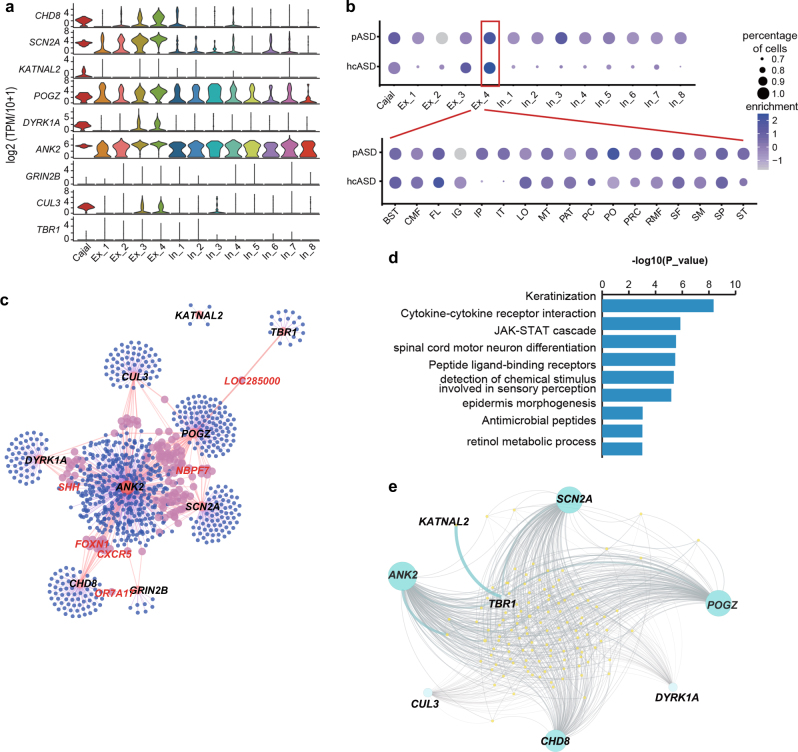
Expression pattern and coexpression networks of autism risk genes. **a** Violin plots showing the epxression levels of the nine hcASD genes in each neuron sub-cluster. **b** Enrichment analysis of the hcASD genes and pASD genes in each neuron sub-cluster and each region of the Ex_4 with neuron cell number over 5. **c** Coexpression network showing the top 1,000 genes coexpressed with the nine hcASD genes. This indicates more candidate ASD genes. **d** Enriched biological processes for genes coexpressed with the nine hcASD genes. **e** The expression network for the hcASD genes and the pASD genes

Autism risk genes might be up-regulated in mid-gestation human cortical projection neurons.^50,51^ As we investigated the transcriptome of the whole cortex at this gestation stage at single-cell resolution, we further analyzed the expression patterns of the high-confidence autism spectrum disorder (hcASD) genes and probable ASD (pASD) genes.^50^ All 9 hcASD genes were detected in the neuronal sub-clusters (Fig. 8a), whereas both hcASD and pASD genes showed expression enrichment in the Ex_4 sub-cluster (Fig. 8b). This finding agreed with previous results suggesting that cortical projection neurons were related to the pathogenesis of autism. We then measured the expression of both sets of ASD genes in Ex_4 in different regions. The pASD genes showed accordant expression across all regions, whereas the hcASD genes showed much lower enrichment scores in the IP and IT regions than those in other regions (Fig. 8b). The co-expression network of these nine hcASD genes revealed a key role for *ANK2* in the expression network, and no interaction of *KATNAL2* with other hcASD genes (Fig. 8c and Supplementary information, Table S5). GO analysis of the co-expressing genes showed the most significant enrichment in keratinization. Other enriched biological processes were revealed, such as spinal cord motor neuron differentiation and epidermis morphogenesis (Fig. 8d). Interaction analysis of the hcASD genes with the pASD genes revealed abundant interactions between *ANK2*, *SCN2A*, and *POGZ* and the pASD genes (Fig. 8e). Both the expression patterns and networks indicated that multiple distinct pathways and processes were potentially involved in the ASD phenotype. In addition to the hcASD and pASD genes, there were more network genes participating in functions that might increase the risk for ASD when they went wrong.

## Discussion

In this study, we uncovered 29 cell sub-clusters within the mid-gestation stage of human embryonic cerebral cortex in vivo and identified the unique signature of each type of cell related to their biological functions, such as in the cell cycle, TF networks, and metabolism. Inhibitory neurons originating from the MGE and the CGE exhibited clear differences in both their gene expression signatures and distribution patterns. The *LHX6*^*+*^ inhibitory neurons tended to be enriched in the top dorsal regions of the cerebral cortex, whereas the *CALB2*^*+*^ ones were in the inferior regions. Our findings indicate the potential in vivo molecular regulation of the maturation process of human excitatory neurons, which provides additional clues to aid in the generation of neurons in vitro with specific physiological functions.

By comparing our single-cell data to those of previous studies, we observed drastic differences between embryonic neurons and adult neurons. The results also indicate that the embryonic excitatory neurons had different gene regulation networks during maturation compared to the inhibitory neurons, and the latter showed delayed maturation compared with the former. For the *SST*^+^ inhibitory neurons, they are the earliest to show up as a subgroup during embryonic development. However, these *SST*^*+*^ neurons are still different from the adult ones as the former showed no maturity apart from the expression of *SST*. It is a very important question of why these *SST*^+^ inhibitory neurons show up earlier than other subtypes. Previous studies proved a key role of inhibitory neurons in circuit development,^52–55^ and thus *SST*^*+*^ inhibitory neurons might play a vanguard role in constructing the local circuit. As different subtypes of inhibitory neurons have different impacts on circuits, for example, the efferents of SST neurons are to principal cells, whereas the efferents of vasointestinal peptide (VIP) interneurons are mainly to SST neurons,^33^ which in turn lead to a disinhibition of those principal cells.^56–58^ It is easy to understand that the VIP neurons are generated later than SST neruons during development as they need local cues from both exicitatory and inhibitory signals for their final positioning^59,60^ and morphological development,^61^ thus SST neurons should show up earlier in the cerebral cortex. Therefore, we infer that the early appearance of *SST*^*+*^ inhibitory neurons at embryonic stage is not to inhibit functions of other neurons as they do in the adult circuits, but these *SST*^*+*^ neurons are helping to form neural circuits. This hypothesis needs more experimental validation in the future.

Regional differences were observed at different aspects. First of all, different cortex regions showed different cell type constitutions, for example, the regions in the inferior surface contain more glia cells, whereas the cortical regions contain more neurons. Moreover, both the excitatory and inhibitory neurons showed asymmetric distributions among the cortical regions. Regions in the frontal lobe contain more excitatory neurons, whereas regions in the temporal lobe contain more inhibitory ones. These differences in cell type constitution could possibly lead to regional specificity of the neural networks and their functions. Besides, neurons of the same sub-cluster showed different degrees of heterogeneity within each region of the cortex, and the frontal lobe was the most homogeneous region for a specific neuron sub-cluster. The different degrees of heterogeneity might be related to the complex neural connections when neurons are fully developed. Moreover, we observed that the maturation of different cortex regions was not synchronized, which may reflect the facts of regional difference in development. At last, we found that even for the same type of neurons, regional specific gene expression patterns existed, and these DEGs were strongly related to known neural diseases or region-specific neural functions.

Neural disorders are affecting numerous patients’ health and life and they are still urgent issues currently without effective solutions. Many of the neural disorders are proved to be connected to the neural developmental stages,^62–66^ and especially the autism spectrum disorder (ASD) was reported to be closely related to the neuron projection at mid-fetal stage in human.^47^ We further analyzed the ASD risk genes in each neuron sub-cluster and brain region, and their network genes were revealed, offering a more precise view of the disease genes’ regulation in the mid-gestation stage of the human cerebral cortex.

In summary, our results contribute to understanding the regionalization of the human cerebral cortex, which lays the ground for dissecting molecular and cellular mechanisms of brain development and diseases.

## Materials and Methods

### Human embryonic brain collection and dissection

Human embryonic brains were obtained from the Third Hospital of Peking University with agreement of the donors. The 22 W and 23 W brains were collected from twins, and we collected each female cerebral cortex from the right hemisphere. We collected the right brain cortex from a 22 W male embryo. Only a few cells were collected from the male cerebral cortex of the right brain at 22 W. For dissection of cortical regions (Fig. 1a and Supplementary information, Table S1), we referred to *The Human Brain during the Second Trimester*, by Shirley A. Bayer.^67^ For each female right brain, we dissected 19 regions from the cerebral cortex, and we also collected the insular gyrus, the pons and the medulla from the inferior surface. We barely cut the germinal zone in each region.

### Tissue digestion and single-cell RNA-seq library preparation

The dissected regions were transferred into 500 µl hibernate E medium (Invitrogen, Cat# A1247601) with 2 mg/ml collagenase IV (Gibco, Cat# 17104-019) and 20 U/µl DNase I (NEB, Cat# M0303L). The samples were roughly pipetted to break down the tissue and then added to 500 µl hibernate E medium containing 1 mg/ml papain (Sigma, Cat# P4762) and 20 U/μl DNase I. The tissue fragments were kept at 37 °C in a thermocycler for 5 min. The sample was then pipetted thoroughly to obtain single cells. To stop digestion, the tubes were centrifuged at 300×* g* for 2 min, the cell pellet was collected, the supernatant was discarded, and the cells were resuspended with 1 ml hibernate E medium. We removed the block mass using a 40-μm filter. The cell suspension was kept on ice to prevent cell death during the single-cell collection procedure. We randomly picked single cells that appeared to be alive and placed them in 2.5 μl cell lysis buffer using a mouth pipet under a microscope. We modified the STRT-seq method for amplification of single-cell transcriptomes by changing the reverse transcription primer, the induced cell barcode, and the unique molecular identifier (UMI). The final primer concentration for reverse transcription was 300 nM. The amplification primers at the 3′ end were chemically modified with biotin. Before library construction, we enriched the fragments containing the cell barcode and UMI with streptavidin beads. The cDNAs of all 96 cells with different cell barcodes were pooled together for one library construction.^68^ Each single cell was sequenced for 2 × 10^6^ of 150-bp paired-end reads using an Illumina HiSeq 4000.

### Processing of single-cell RNA-Seq data

Raw reads were first segregated based on the cell-specific barcode information in read 2 of the pair-ended reads. Then, sequences in read 1 were trimmed with customized scripts to remove the TSO sequence, the polyA tail sequence and sequences with low-quality bases (N > 10%) or contaminated with adapters. Subsequently, the stripped read 1 sequences were aligned to the hg19 human reference genome (UCSC) using TopHat (version 2.0.12).^69^ Uniquely mapped reads were counted using htseq-count from the HTSeq package^70^ and then grouped based on the cell-specific barcodes. For each gene, we discarded duplicated transcripts with identical UMIs. Finally, the transcript number for each gene in each cell was quantified by the number of distinct UMIs of that gene.

In total, we sequenced 4,664 single cells, and cells with fewer than 1,000 detected genes, 20,000 detected transcripts and 20% mapping ratio were removed. We also removed cells with too many raw reads, as these cells may not truly be single cells, thus leaving 4,233 cells for further analysis. Because most of our single cells did not reach one million UMIs, we normalized the expression value by log_2_(TPM/10 + 1) rather than log_2_(TPM + 1) (TPM: transcripts per million). By doing so, we avoided counting each transcript several times and overestimating the gene expressions.

### Nonlinear dimensional reduction (t-SNE) and clustering

We visualized our 4,233 single cells by t-SNE using the Barnes-Hut algorithm (implemented in the Rtsne package in R). First, we used the Seurat method to select the highly variable genes (HVGs) based on the log_2_(TPM/10 + 1) expression values. Only genes with an expression level >1 and expressed in at least 3 single cells were considered, whereas single cells with <1,000 expressed genes were excluded, leaving 4,213 cells for the subsequent analysis. HVGs with an average expression greater than 1 and a dispersion greater than 1 were used as inputs for the t-SNE analysis.

To cluster the cells, we first used the Seurat Find Clusters function, which is an implementation of a graph-based clustering approach, to obtain clusters based on all the HVGs (for details, see http://satijalab.org/seurat/pbmc-tutorial.html). We merged the obtained clusters into three main groups, namely, a neuron group, a glial cell group, and a non-neural cell group (see Supplementary information, Figure S1a). Second, as the heterogeneity of fetal brain cells was limited, to further obtain accurate subgroups in each main group, we employed previously reported methods.^26^ For each iteration, this method can split a certain group into 2 subgroups. We could then decide whether the obtained subgroups should be separated into smaller subgroups based on the feature genes selected by the random forest algorithm and the DEGs of the obtained subgroups. Specifically, subgroups were classified and verified using unsupervised hierarchical clustering and a random forest algorithm in R. In brief, (1) we first calculated the gene expression variation for each gene across all cells as CV^2^ = variance/mean^2^, which was then fitted to an inverse distribution, and we chose genes with a CV^2^ beyond one standard error of the mean; (2) we then carried out hierarchical clustering and determined two clusters at the first split; (3) we performed a 10-fold random forest feature selection to select DEGs dividing the two clusters; (4) for each class, we selected samples with internal vote probabilities > 0.6 as the training set to achieve an optimal classifier, which was used to predict the rest of the samples; (5) 100 runs of 10-fold random forest cross-validation (CV) were carried out, and the samples with internal vote probabilities < 0.55 were abandoned; (6) we repeated steps 1–5 on the newly formed classes to obtain finer clusters. Finally, 13 clusters, including 8 inhibitory neuron clusters, 4 excitatory neuron clusters, and 1 Cajal-Retzius cell cluster, were identified for the neuron group (see Figs. 1a and 2a), 6 clusters for the glial cell group (see Supplementary information, Figure S3a) and 10 clusters for the non-neural cell group (see Supplementary information, Figure S4a). In addition, subsequent t-SNE plots for each primary group were generated by the Rtsne package using the DEGs (see below) of all the clusters in that group, except in the neuron group, for which we did not include the Cajal-Retzius cell cluster.

### Identification of DEGs and GO analysis

To identify unique cluster-specific marker genes, we used the Seurat function find_all_markers (thresh.test = 1, test.use = “roc”). For two given clusters, the find.markers function was used to identify DEGs with the parameters thresh.use=1, test.use=“roc”. In the roc test, a value representing the ‘classification power’ ranging from 0 (for ‘random’) to 1 (for ‘perfect’) would be generated for a certain gene. We chose genes with a fold-change ≥ 2 or ≤ 0.5 and a power ≥ 0.4 as DEGs. The R software program was used to plot heatmaps, pie charts, and bar plots. Violin plots were generated using Seurat. Enrichment analysis was performed using Metascape (http://metascape.org).^71^ Gene information involved in certain pathways was selected from KEGG (http://www.genome.jp/kegg/pathway.html).^72^

### Developmental pseudotime analysis

The monocle2 package in R^73,74^ was used to determine the developmental pseudotime of excitatory neurons and glial cells. Following the monocle vignette, we used UMI count data as input and selected DEGs identified in the last procedure to order the cells. For all other parameters, the default settings were used. We also plotted eight TFs in DEGs along the inferred developmental pseudotime. The regulation networks for the 8 TFs were constructed by GENIE3 package and plotted by cytoscape.

For excitatory neurons, in Fig. 2e and Supplementary information, Figure S2 (right), we compared our dataset with two 21 pcw datasets from Miller et al.^20^ to see in which regions the subtype marker genes were enriched. We downloaded the microarray datasets and extracted the intersection genes with the genes identified to show regulation in excitatory neuron differentiation by monocle2. We then plotted heatmaps using *z*-score values with genes ordered as in Fig. 2e (left) and samples ordered from inner to outer layers of the prenatal neocortex.

### Cell cycle analysis

A previously reported core gene set was used to perform cell cycle analyses, including 43 G1/S and 54 G2/M genes.^75,76^ We calculated the average expression of each gene set as the corresponding score. Cells were determined to be quiescent if their G1/S score < 2 and G2/M score < 2; otherwise, they were deemed proliferative. In addition, proliferative cells were designated G2/M if their G2/M score > G1/S score, whereas cells were designated G1/S if their G1/S score > G2/M score.

### *LHX6*^*+*^ interneuron and *CALB2*^*+*^ interneuron ratio in each specified cerebral cortex region

For each region, we defined the interneuron cells with a log_2_(TPM of *LHX6*/10 + 1) greater than 1 as *LHX6*^*+*^ interneurons. Similarly, interneuron cells with a log_2_(TPM of *CALB2*/10 + 1) greater than 1 were defined as *CALB2*^*+*^ interneurons. Then, the *LHX6*^*+*^ interneuron ratio was calculated as the number of *LHX6*^*+*^ interneurons to the total number of *LHX6*^*+*^ interneurons and *CALB2*^*+*^ interneurons. In addition, *LHX6*^*+*^ regions were recognized as regions with a *LHX6*^*+*^ interneuron ratio > 0.6. *CALB2*^*+*^ regions were identified in the same way. Regions that contained each type of interneuron between 0.4 and 0.6 were considered balanced regions. We did not include regions with a sum of both types of neurons less than 15.

### Comparison with previous published datasets

To reveal more developmental clues, we compared our dataset with three published datasets of the human cortex: two fetal^11,27^ and one adult dataset.^26^ We combined our dataset with the two fetal datasets using the mnnCorrect function of the scran package in R.^37,38^ This strategy relied on a shared subset of the population between batches and detected MNNs in the high-dimensional expression space to correct batch effects. The corrected dataset was then used to perform PCA through the FactoMineR package in R. Due to the abundance of cells in our dataset, there would have been PCA bias if we had used all the cells. Thus, we randomly selected 200 excitatory and 200 interneuron cells to redo the PCA. For each of the first four PC dimensions, the top 50 correlated genes combining both positively and negatively correlated genes were selected. We then performed unsupervised hierarchical clustering using Pearson correlation based on these genes through hclust in R. Cluster-specific markers were identified by *t*-test, and the *P*-value was corrected by the BH method using the p.adjust function.

For comparison with the adult cortex dataset, we first used Seurat to identify HVGs with an average expression > 1 and dispersion > 1 for both the adult dataset and our dataset separately. The expression value of the adult dataset was normalized by log_2_(TPM + 1) as used in the paper that it came from, while the expression value of our dataset was normalized by log_2_(TPM/10 + 1). Then, we calculated the *z*-score values for both datasets based on their own HVGs. Finally, we combined the two z-score-based datasets using all the HVGs to perform PCA through FactoMineR package in R. For each of the first four PCs, genes with correlation > 0.3 or < –0.3 were selected to plot heatmaps and to perform enrichment analysis. However, for the PC1 positive direction, we chose the top 100 correlated genes because there were too many genes whose correlation exceeded 0.3.

### Enrichment score for autism gene sets and gene regulation network

To study the expression pattern of ASD-related genes, we collected two datasets described in Willsey et al.: 9 high-confidence ASD (hcASD) genes and 122 probable ASD (pASD) genes.^50^ We calculated the enrichment score for these two gene sets across all neuron cells using the AUCell package (https://github.com/aertslab/GENIE3).^77,78^ Regulation networks for the 9 hcASD genes were constructed by the GENIE3 package (https://github.com/aertslab/AUCell)^45^ and plotted by Cytoscape.

### Regional heterogeneity of neurons across the whole cerebral cortex

For frontal, parietal, temporal, and occipital lobes, and inferior regions, we explored the regional heterogeneity of neurons by calculating the Pearson correlation between single cells of the heterogeneous genes for each sub-cluster whose cell number was > 200. For each sub-cluster, we used Seurat to identify HVGs with an average expression > 1 and a dispersion > 1. We then calculated the Pearson correlation using these HVGs. For cells of each sub-cluster in a certain region, we first obtained the correlation distribution of single cells in that region, excluded self-correlation values (for which the value should be 1) and then calculated the correlation with cells in each of the other four regions. Wilcox.test in R was performed to test the significance of correlation between two distributions. To ensure that the conclusion was valid, we also identified HVGs with an average expression > 1 and dispersion > 0.5 to calculate the Pearson correlation and tested the significance of correlations between two distributions. The results of this procedure were similar (data not shown).

### Developmental maturation degree across the whole cortex

We used two independent methods to estimate the developmental maturation degree across the whole cortex. The first method evaluated synapse formation and function across cortical regions. The rationale was that a region is believed to be more mature if there are more cells that have formed synapses or whose synapses are functional. Thus, we calculated the mean expression of the gene set of GO terms 0007268-0007271 and 0016079-0016082 for each neuron (including both excitatory and inhibitory neurons) and averaged the expression values for a certain region, which was regarded as an indicator of synapse formation and function for that region. In addition, regions with fewer than 50 excitatory neurons were excluded. We then displayed the values of the cortical regions using heatmaps (see Fig. 4e).

The second method estimated the ratio of excitatory neurons expressing *CUX2*. Because *CUX2* is an upper layer marker and cortical development follows an inside-out order for layer formation, a region is more mature if there are more excitatory neurons expressing *CUX2*. In this analysis, only excitatory neurons whose *CUX2* expression value based on the log_2_(TPM/10 + 1) was > 1 were deemed to exhibit true expression. Thus, for a certain region, the ratio of excitatory neurons expressing *CUX2* was calculated as excitatory neurons expressing *CUX2/*all excitatory neurons. In addition, regions with fewer than 50 excitatory neurons were excluded. We also displayed these cortical region values using heatmaps (see Fig. 4c).

### RT-qPCR validation

We dissected the IG, IT, PC, SP regions when we received the brain sample of a 22 W female embryo. Each region was digested into cell suspensions before extracting the total RNA with QIAGEN RNeasy mini kit (QIAGEN, Cat# 74104). Then reverse transcription was carried out in a volume of 19 μl, containing 2.5 ng/μl oligod(T) primer (Takara, Cat# 3806), 0.5 mM dNTP mixture (Takara, Cat# R045B), 2 U/μl RNase inhibitor (Ambion, Cat# AM2684), 5 mM DTT, 1× first strand and 0.5 U/μl superscript III reverse transcriptase (Invitrogen, Cat# 18080-044). After reverse transcription at 50 °C for 60 min and 70 °C for 15 min to inactivate the enzyme, 1 μl RNase H (Invitrogen, Cat# 18021-071) was added into each reaction tube to digest RNA. For each sample we added nuclease-free water to obtain 1 ng/μl cDNA. The qPCR detection was run on CFX connect system (Biorad) using SYBR Green (Roche, Cat# 13396700). Primers for each genes: GAPDH forward, CGACACCCACTCCTCCACCT, reverse, CTTGTGCTCTTGCTGGGGCT; NRGN forward, AGCGTCACCCAAGCACACTC, reverse, GCAAGGGTCGTCCGAAACCA; GAD1 forward, CGCTCTCTGTCTGGCTGTACG, reverse, ACAGTTGTGAGCCTGGTCACTT; NEUROD2 forward, GGTTCCCCCAAAAAGGGGCA, reverse, GGGTGTCCGACGGGAGTTTC.

### Immunohistochemistry

We fixed tissue samples in 4% paraformaldehyde for 16 h, cryoprotected them in 30% sucrose, and embedded them in optimal cutting temperature compound (Thermo Scientific). Then, 40-μm cryosections were collected on Superfrost slides (VWR) using a Leica CM3050S cryostat. Primary antibodies rabbit anti-NEUROD2 (1:500, Abcam ab104430), mouse anti-TNNT2 (1:100, Abcam ab8295), rabbit anti-GFAP (1:250, Sigma G9269) were diluted in blocking buffer containing 10% donkey serum, 0.5% Triton X-100 and 0.2% gelatin diluted in PBS at pH 7.4. Binding was revealed using an appropriate Alexa FluorTM 488, Alexa FluorTM 594, or Alexa FluorTM 647 fluorophore-conjugated secondary antibody (Life Technologies). Cell nuclei were counter-stained using DAPI (Life Technologies). Images were collected using an Olympus FV1000 confocal microscope.

### In situ hybridization

Probes complementary to target human mRNAs used for RNA in situ hybridization were cloned from primary human fetal cortical cDNA reverse-transcribed using PrimeScript II 1st Strand cDNA Synthesis Kit (Takara) with oligo dT primers. The RNA samples were isolated from 22 W and 23 W human cortex using SV Total RNA Isolation System (Promega). Specific genes were amplified using the following primers: RAMP3 forward, AAG GCT TTC GCA GAC ATG AT, reverse, ACA GGA TGC AGC AGG TGA TT; PTGDS forward, GCT CCT CCT GCA CAC CTC, reverse, CAA TGG TAT CCT CTG TGA AGC CC; CUX2 forward, CTG GAG AAG AAA GCC TAC CT, reverse, GAC AGG TGA CAC AGA CAT CAT G. Primers specific to target genes of interest were designed using Primer3. PCR used Q5 High-Fidelity DNA Polymerase (NEB). PCR products of predicted band sizes were gel-extracted and ligated using the Hieff CloneTM Plus One Step Cloning Kit (Yeason). Ligation products were transfected into Trans5α Chemically Competent *E. coli* (Transgene). Cloned sequences were confirmed by sequencing. Digoxigenin-labeled RNA probes for in situ hybridization were generated by linearizing the pSPT18 Vector and in vitro-transcribing the probe using T7 or SP6 RNA Polymerase (Roche) in the presence of DIG-RNA Labeling Mix (Roche). In situ hybridization was performed blinded to the sense/antisense status of each probe, and sense control probes gave no signal (data not shown). The in situ hybridization protocol was described previously (Inma Cobos).

### Data and software availability

The accession number for all sequencing data reported in this paper is GEO: GSE103723 (The following secure token has been created to allow review of record GSE103723 while it remains in private status: wnyxaammzloppsl). The expression pattern of our single-cell RNA-seq data are available from the corresponding author upon request.

## Electronic supplementary material

Supplementary information, Figure S1

Supplementary information, Figure S2

Supplementary information, Figure S3

Supplementary information, Figure S4

Supplementary information, Figure S5

Supplementary information, Figure S6

Supplementary information, Table S1

Supplementary information, Table S2

Supplementary information, Table S3

Supplementary information, Table S4

Supplementary information, Table S5
