# Supplementary material for: Spatial transcriptomic survey of human embryonic cerebral cortex by single-cell RNA-seq analysis
Source: Cell Res. 2018 Jun 4;28(7):730–45. doi: 10.1038/s41422-018-0053-3 (PMC6028726; doi:10.1038/s41422-018-0053-3)
Supplement: Supplementary file 1 — Supplementary information, Figure S1 [file 41422_2018_53_MOESM1_ESM.pdf]

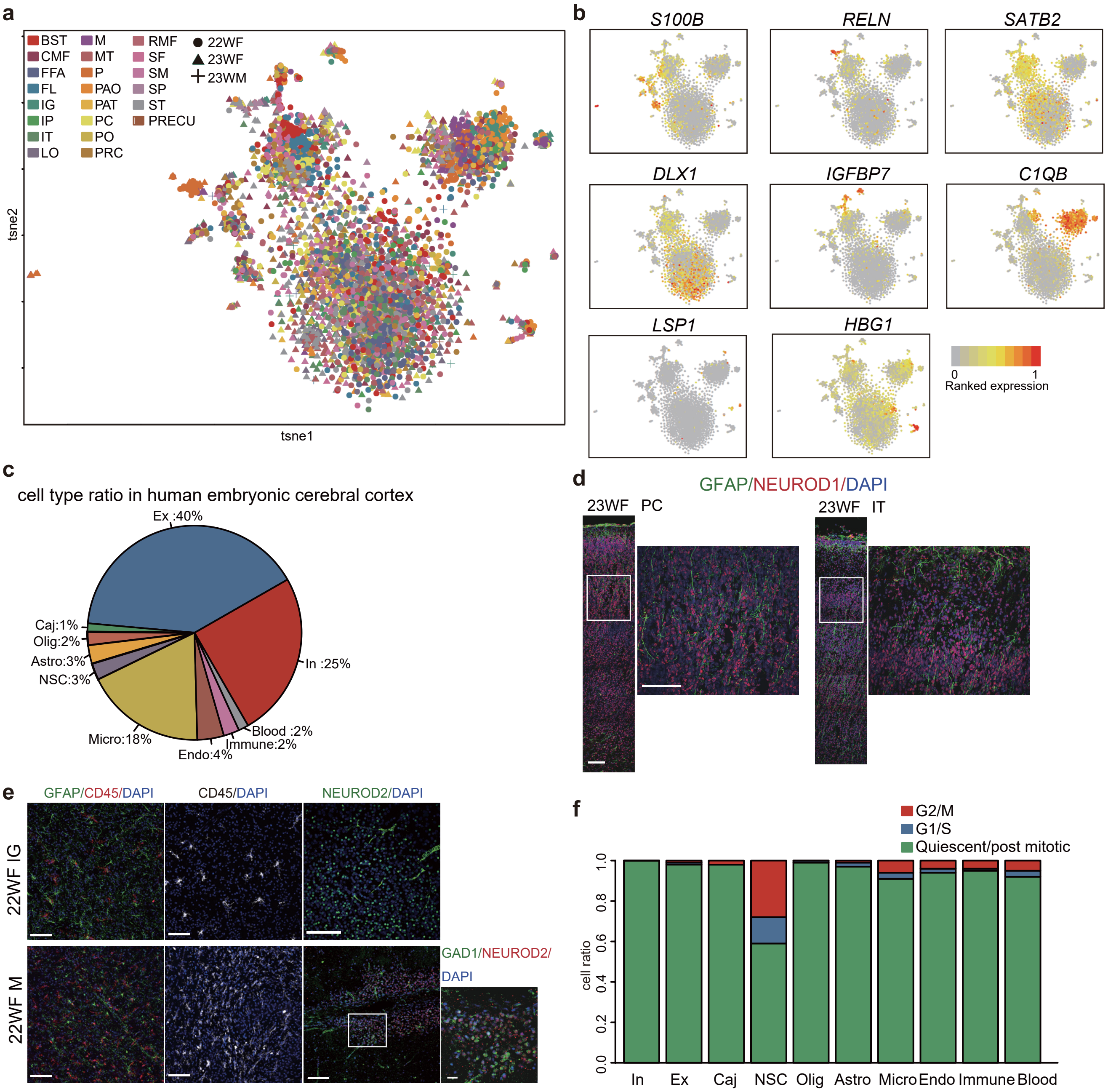

Supplementary Figure 1. Global characters of each cell type in the human embryonic cerebral cortex

(a) T-SNE map showing the clustering of cells indicates almost no batch effect on different embryo sample or regions. (b) Dot plots showing the marker genes' expression of each cell type identified in Fig. 1a. The expression levels were normalized to 0 to 1 according to the TPM value across all cells of one gene. (c) Cell type ratios on the whole developing cortex. (d) Immunostaining of NEUROD1 and GFAP in PC and IT region clearly show more neurons in the cortex at this stage than glia. The scale bar shows 100 μm. (e) Immunostaining of astrocytes, microglias, excitatory neurons and inhibitory neurons in the inferior surface regions. IG, insular gyrus; M, medulla. The scale bar shows 100 μm except 20 μm for the zoomed in. (f) Histogram of cell ratios in different cell cycle stages inside each type.
