# Supplementary material for: Spatial transcriptomic survey of human embryonic cerebral cortex by single-cell RNA-seq analysis
Source: Cell Res. 2018 Jun 4;28(7):730–45. doi: 10.1038/s41422-018-0053-3 (PMC6028726; doi:10.1038/s41422-018-0053-3)
Supplement: Supplementary file 2 — Supplementary information, Figure S2 [file 41422_2018_53_MOESM2_ESM.pdf]

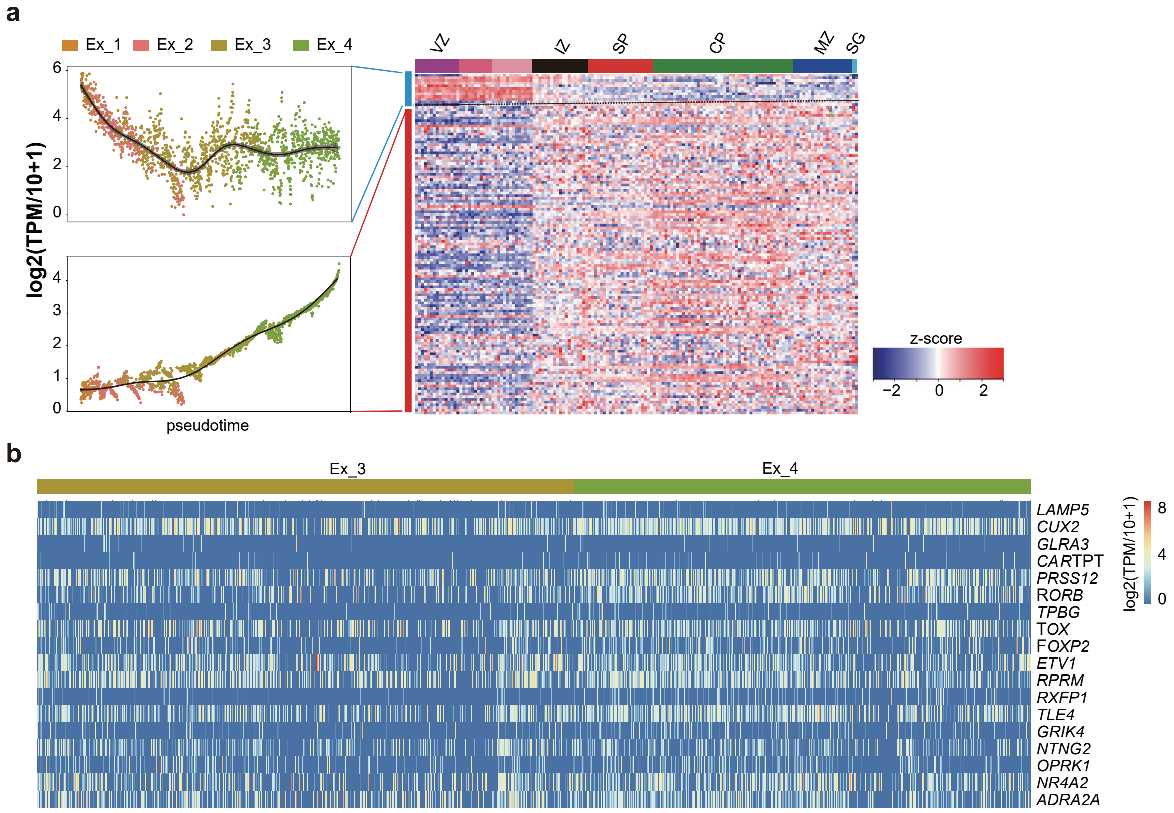

Supplementary Figure 2. Pseudotime regulation genes analysis and layer markers' expressions for excitatory subclusters
(a) Putative excitatory neuron maturation regulating genes' expression in the structures studied by Miller. et al.(2014) (ref 11, sample 12690). Also see Fig. 2e. (b) Heatmap showing the layer markers' expressions in each single cell of Ex_3 and Ex_4 subclusters. Most cells co-express multiple layers' marker genes at embryonic stage.
