# Supplementary material for: Spatial transcriptomic survey of human embryonic cerebral cortex by single-cell RNA-seq analysis
Source: Cell Res. 2018 Jun 4;28(7):730–45. doi: 10.1038/s41422-018-0053-3 (PMC6028726; doi:10.1038/s41422-018-0053-3)
Supplement: Supplementary file 3 — Supplementary information, Figure S3 [file 41422_2018_53_MOESM3_ESM.pdf]

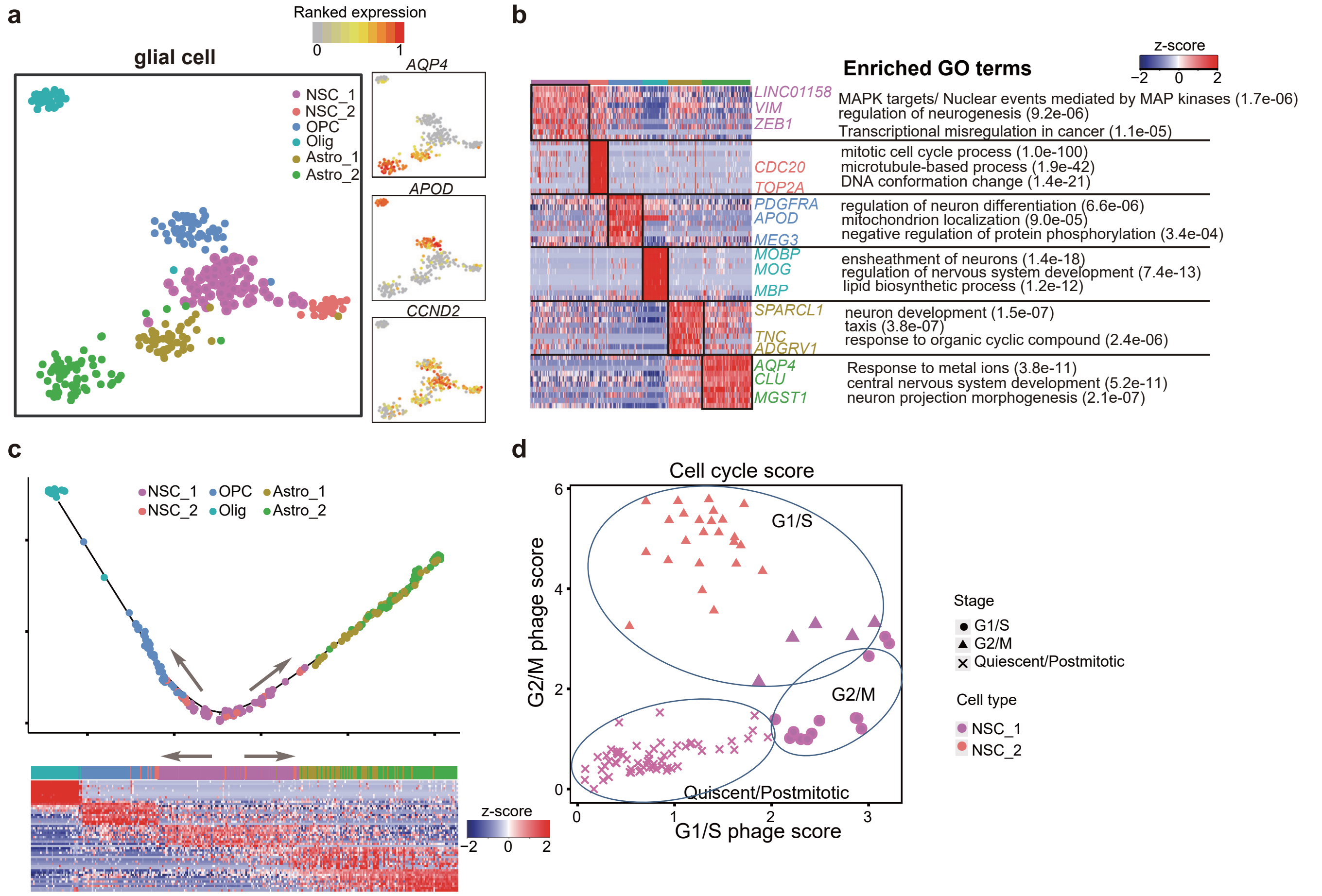

Supplementary Figure 3. Glial cell subclusters in the developing cerebral cortex

(a) TSNE shows the subclusters of glia cell including NSC, astrocyte, OPC and oligdendrocyte. Dotplots on the right show the typical cell type markers' expressions. (b) Heatmap of subcluster specific genes and their corresponding enriched biological processes. (c) Pseudotime analysis shows a differentiation routine of NSCs to oligdendrocytes and NSCs to astrocytes, respectively. Also the genes participate in the differentiation process are drew out as a heatmap at the bottom. (d) Cell cycling stage analysis of the two subgroups of NSCs.
