# Supplementary material for: Spatial transcriptomic survey of human embryonic cerebral cortex by single-cell RNA-seq analysis
Source: Cell Res. 2018 Jun 4;28(7):730–45. doi: 10.1038/s41422-018-0053-3 (PMC6028726; doi:10.1038/s41422-018-0053-3)
Supplement: Supplementary file 4 — Supplementary information, Figure S4 [file 41422_2018_53_MOESM4_ESM.pdf]

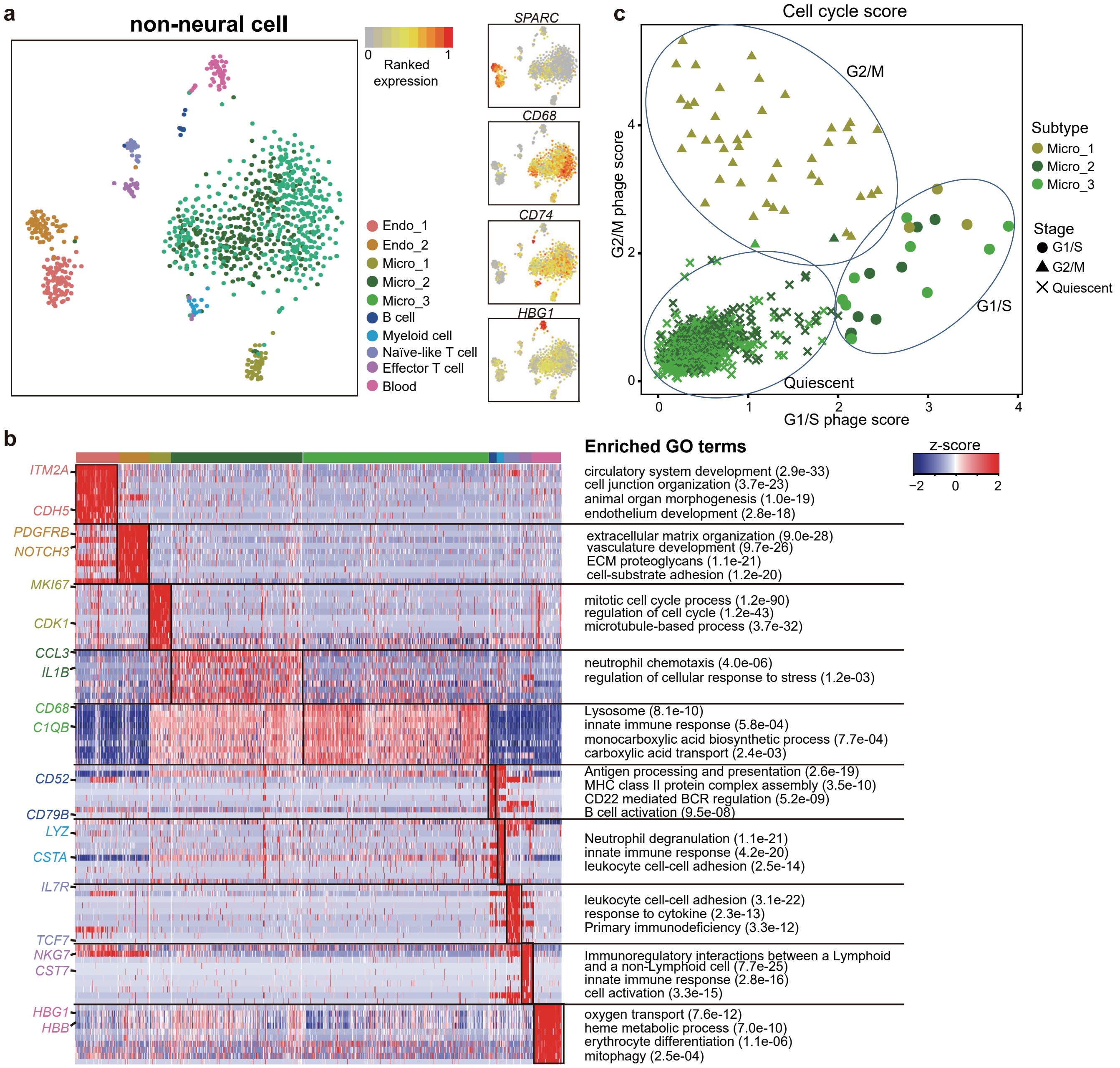

Supplementary Figure 4. Subclusters of non-neural cells in the developing cerebral cortex

(a) TSNE shows the subclusters of cells which are not neural origination including microglia, endothelial cell, immune cell and blood cell. Dotplots on the right show the typical cell type markers' expressions. (b) Heatmap of subcluster spcific genes and their corresponding enriched biological processes. (c) Cell cycling stage analysis of the three subclusters of microglias.
