# Supplementary material for: Spatial transcriptomic survey of human embryonic cerebral cortex by single-cell RNA-seq analysis
Source: Cell Res. 2018 Jun 4;28(7):730–45. doi: 10.1038/s41422-018-0053-3 (PMC6028726; doi:10.1038/s41422-018-0053-3)
Supplement: Supplementary file 5 — Supplementary information, Figure S5 [file 41422_2018_53_MOESM5_ESM.pdf]

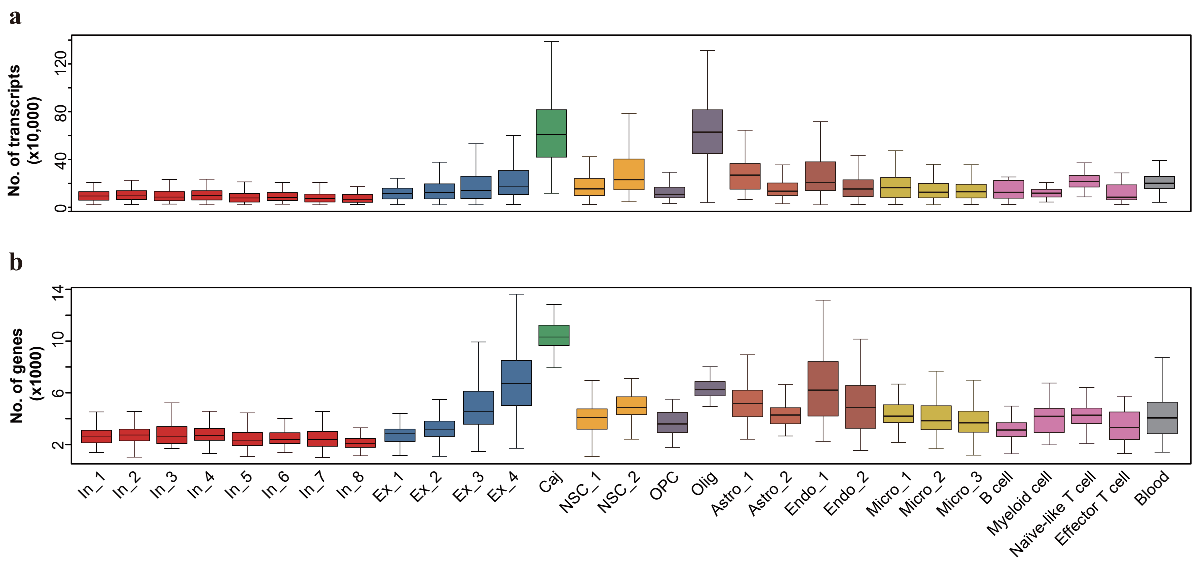

Supplementary Figure 5. Global characters of each subcluster

Boxplots show the number of transcripts (a) and genes (b) for single cells in each subgroup.
