# Supplementary material for: Spatial transcriptomic survey of human embryonic cerebral cortex by single-cell RNA-seq analysis
Source: Cell Res. 2018 Jun 4;28(7):730–45. doi: 10.1038/s41422-018-0053-3 (PMC6028726; doi:10.1038/s41422-018-0053-3)
Supplement: Supplementary file 6 — Supplementary information, Figure S6 [file 41422_2018_53_MOESM6_ESM.pdf]

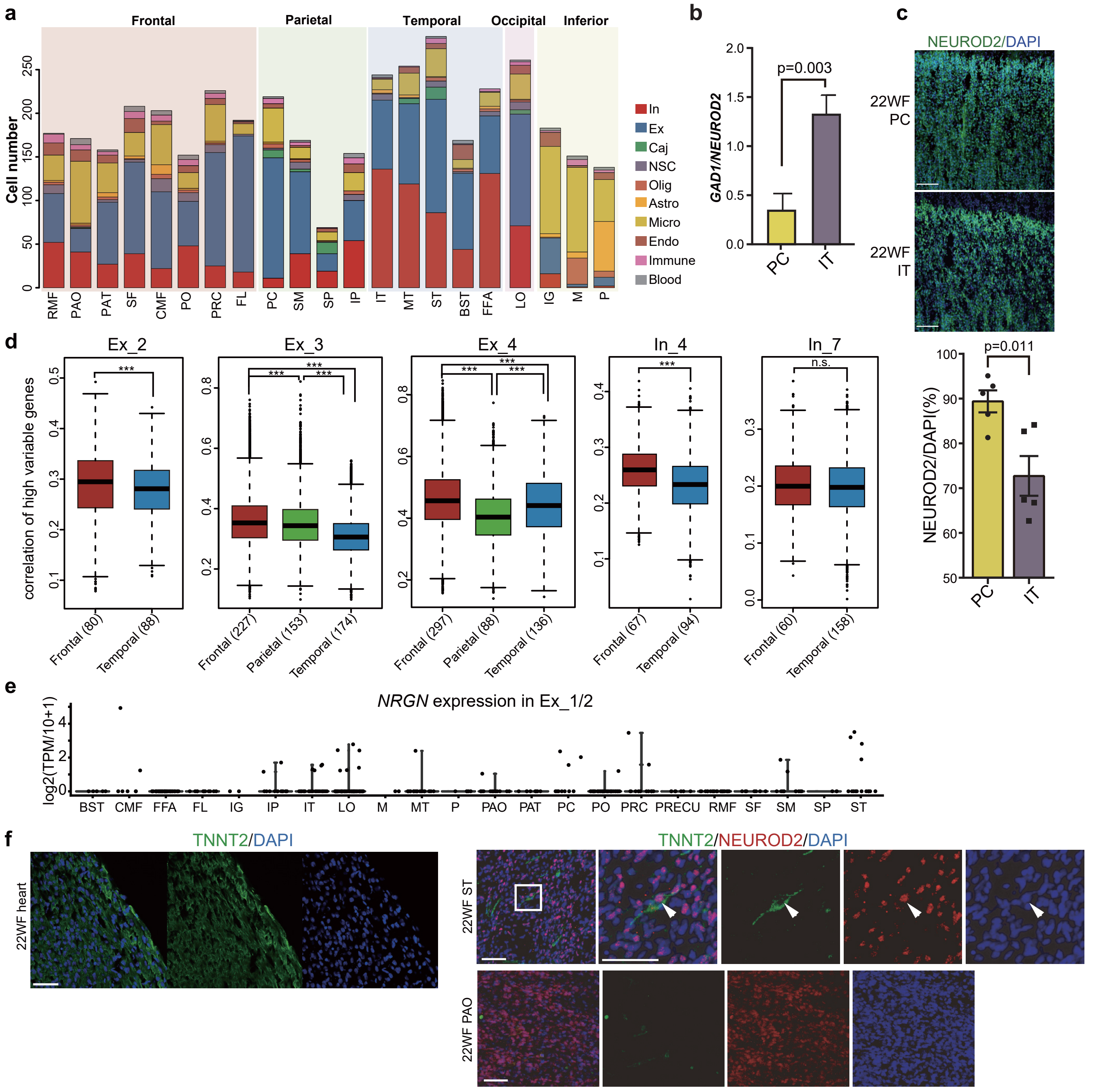

Supplementary Figure 6. Cell type diversities and cell heterogenity in regions of the human developing cortex
(a) Barplot showing the cell numbers of all cell types in each region. (b) RT-QPCR of *NEUROD2* and *GAD1* in PC and IT regions shows higher expression of *GAD1* in IT region while the PC regions expressed higher level of *NEUROD2*. (c) Immunostaining of NEUROD2 in PC and IT regions and the histogram shows the statistics of NEUROD2 positive cells in each region. The PC region shows relative higher percentage of NEUROD2 positive cells. The scale bar shows 100 μm (d) Boxplots showing the correlation coefficients of the heterogenous genes with disperse over 1 between single cells of the same subtype inside each region. For each subtype, the number of cells observed in each region was shown in the bracket. '*' stands for p_value between 1e-4 and 1e-8; '**' stands for p_value between 1e-8 and 1e-12; '***' stands for p_value less than 1e-12; 'n.s.' stands for p_value over 1e-4. (e) boxplot showing the sparse expression of NRGN in Ex_1/2 among the regions. (f)Immunofluorescence of TNNT2 in the cardiomyocytes from a 22WF heart tissue (top). Validation of excitatory neurons expressing myocardial protein TNNT2 in the 22WF ST region by immunofluorescence. The PAO region is displayed as a negative control. The scale bar shows 50 μm.
