# Supplementary material for: Spatial transcriptomic survey of human embryonic cerebral cortex by single-cell RNA-seq analysis
Source: Cell Res. 2018 Jun 4;28(7):730–45. doi: 10.1038/s41422-018-0053-3 (PMC6028726; doi:10.1038/s41422-018-0053-3)
Supplement: Supplementary file 7 — Supplementary information, Table S1 [file 41422_2018_53_MOESM7_ESM.pdf]

brain region	abbr.	belonging	GW22F cell number		GW23F cell number		GW23M cell number		Total cell number	
			Quality-passed	amplified	Quality-passed	amplified	Quality-passed	amplified	Quality-passed	amplified
rostral-middle-frontal	RMF	Frontal lobe	82	96	107	136	0	0	189	232
pars orbitalis	PAO		92	96	82	96	0	0	174	192
pars triangulars	PAT		81	96	88	88	0	0	169	184
superior frontal	SF		80	96	144	168	0	0	224	264
caudal-middle-frontal	CMF		95	96	125	136	0	0	220	232
pars opercularis	PO		77	96	90	96	0	0	167	192
pre-central	PRC		92	96	140	144	0	0	232	240
frontal lobe	FL		181	192	0	0	15	16	196	208
post-central	PC	Parietal lobe	95	96	129	136	0	0	224	232
supra-maginal	SM		86	96	90	96	0	0	176	192
superior parietal	SP		0	0	75	80	0	0	75	80
inferior parietal	IP		78	96	89	96	0	0	167	192
precuneus	PRECU		0	96	0	0	21	24	21	120
inferior temporal	IT	Temporal lobe	87	96	170	200	0	0	257	296
middle temporal	MT		81	96	184	192	0	0	265	288
superior temporal	ST		84	96	214	224	0	0	298	320
bank superior temporal	BST		178	192	0	0	0	0	178	192
fusiform	FFA		161	192	45	48	24	24	230	264
lateral occipital	LO	Occipital lobe	88	96	177	184	0	0	265	280
insular gyrus	IG	Inferior surface	89	96	86	88	20	24	195	208
medulla	M		86	96	70	80	0	0	156	176
pons	P		83	96	72	88	0	0	155	184
**Total**	——	——	**1,976**	**2,304**	**2,177**	**2,376**	**80**	**88**	**4,233**	**4,768**

Supplementary Table S1 Cell collection information from regions of each sample
